# Bioproduction of Linalool From Paper Mill Waste

**DOI:** 10.3389/fbioe.2022.892896

**Published:** 2022-05-30

**Authors:** Mauro A. Rinaldi, Shirley Tait, Helen S. Toogood, Nigel S. Scrutton

**Affiliations:** ^1^ Future Biomanufacturing Research Hub, Manchester, United Kingdom; ^2^ Manchester Institute of Biotechnology, The University of Manchester, Manchester, United Kingdom; ^3^ C3 Biotechnologies (Maritime and Aerospace) Ltd, Lancaster, United Kingdom

**Keywords:** terpenoids, biomanufacturing, paper mill waste, cellulose, linalool, high-value chemicals, plasmids, synthetic biology

## Abstract

A key challenge in chemicals biomanufacturing is the maintenance of stable, highly productive microbial strains to enable cost-effective fermentation at scale. A “cookie-cutter” approach to microbial engineering is often used to optimize host stability and productivity. This can involve identifying potential limitations in strain characteristics followed by attempts to systematically optimize production strains by targeted engineering. Such targeted approaches however do not always lead to the desired traits. Here, we demonstrate both ‘hit and miss’ outcomes of targeted approaches in attempts to generate a stable *Escherichia coli* strain for the bioproduction of the monoterpenoid linalool, a fragrance molecule of industrial interest. First, we stabilized linalool production strains by eliminating repetitive sequences responsible for excision of pathway components in plasmid constructs that encode the pathway for linalool production. These optimized pathway constructs were then integrated within the genome of *E. coli* in three parts to eliminate a need for antibiotics to maintain linalool production. Additional strategies were also employed including: reduction in cytotoxicity of linalool by adaptive laboratory evolution and modification or homologous gene replacement of key bottleneck enzymes GPPS/LinS. Our study highlights that a major factor influencing linalool titres in *E. coli* is the stability of the genetic construct against excision or similar recombination events. Other factors, such as decreasing linalool cytotoxicity and changing pathway genes, did not lead to improvements in the stability or titres obtained. With the objective of reducing fermentation costs at scale, the use of minimal base medium containing paper mill wastewater secondary paper fiber as sole carbon source was also investigated. This involved simultaneous saccharification and fermentation using either supplemental cellulase blends or by co-expressing secretable cellulases in *E. coli* containing the stabilized linalool production pathway. Combined, this study has demonstrated a stable method for linalool production using an abundant and low-cost feedstock and improved production strains, providing an important proof-of-concept for chemicals production from paper mill waste streams. For scaled production, optimization will be required, using more holistic approaches that involve further rounds of microbial engineering and fermentation process development.

## Introduction

There needs to be a global transition from an overreliance on petrochemical feedstocks, polluting chemical processes and non-sustainable practices towards green biomanufacturing using microorganisms to access renewable carbon sources (Lv et al., 2020). A surge in the development of green biomanufacturing technologies over the past decade, in part realized through the engineering of biology, has encompassed diverse applications within the biopharmaceutical, energy, chemicals, and advanced synthetic fuels sectors. This interest has led to the use of robust industrial microbial hosts, *de novo* pathway constructions for new and existing chemicals production and utilization of renewable and sustainable carbon sources as feedstocks (Lv et al., 2020). Biomanufacturing routes have the potential to make virtually any chemical from biogenic waste feedstocks. This avoids reliance on petrochemical feedstocks, thus contributing towards net zero carbon emissions for chemicals manufacturing (Isikgor and Becer, 2015). However, technical barriers remain to implementing biomanufacturing routes at scale. These include low production titers and high operational costs of scaled fermentations relative to synthetic chemicals production or sourcing of chemicals from agricultural/natural sources.

Terpenoids are an extensive and diverse group of natural and semisynthetic chemicals with biological/medical and industrial applications within the pharmaceutical, fuels and/or chemicals industries (Tetali, 2019). Production of these often-high value chemicals through the engineering of biology has been studied extensively, with each final product derived from common precursor pathways, such as the mevalonate (MVA) or 2-C-methyl-D-erythritol 4-phosphate (MEP) precursor upregulation pathway(s) (Martin et al., 2003). Enantiopure monoterpenoids can be produced to g/L levels using *Escherichia coli* as the microbial host (Rinaldi et al., 2021), which is often higher than using other microorganisms (Moser and Pichler, 2019). One high value monoterpenoid is the natural product (*R*)-linalool, an odoriferous acyclic compound widely used as a scent in cosmetic and household cleaning products (Ferraz et al., 2021). Linalool has pharmaceutical applications as a precursor for vitamin E synthesis. It has also been identified as a precursor molecule for the production of sustainable jet fuels (Gupta and Phulara, 2015; Ferraz et al., 2021). The global linalool consumption is ∼21,000 metric tonnes per annum, with prices ranging from US$6/kg for racemic mixtures from chemical synthesis to US$18/kg for high-end enantiopure linalool extracted from natural sources (QYR Chemical & Material Research Center, 2017).

**FIGURE 1:**
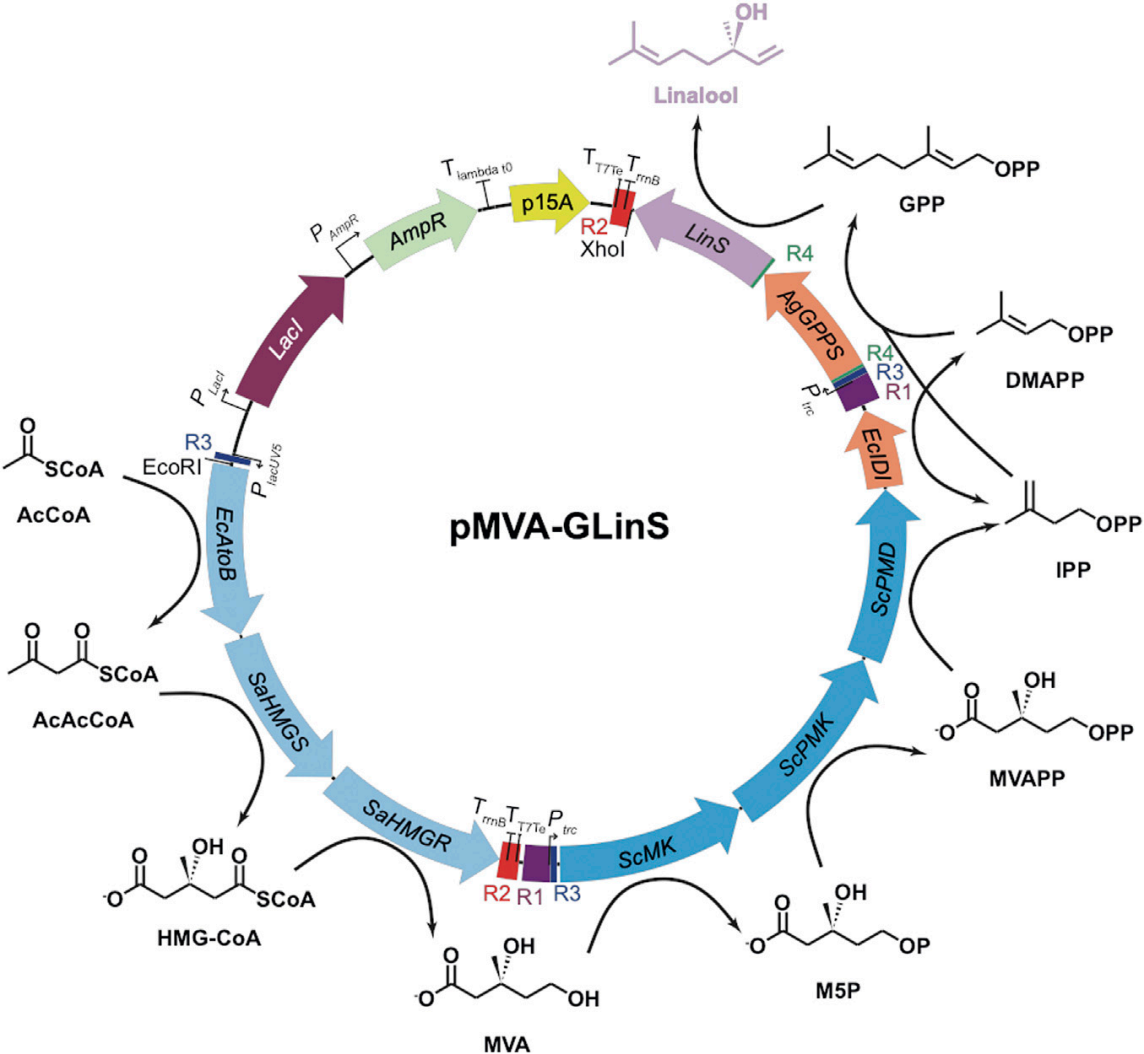
Recombinant pathway for linalool production in *E. coli* (Leferink et al., 2016; Ferraz et al., 2021). Upper (MevT) and lower (MevB) MVA pathway enzymes are shown in light and dark blue, respectively (Alonso-Gutierrez et al., 2013). Enzymes: AgGPPS = geranyl pyrophosphate synthase; EcAtoB = acetoacetyl-CoA thiolase; EcIDI = isopentenyl diphosphate isomerase; LinS = linalool synthase; SaHMGR = HMG-CoA reductase; SaHMGS = HMG-CoA synthase; ScMK = mevalonate kinase; ScPMD = phosphomevalonate decarboxylase and ScPMK = phosphomevalonate kinase. Chemicals: AcAcCoA = acetoacetyl-CoA; AcCoA = acetyl-CoA; DMAPP = dimethylallyl diphosphate; GPP = geranylpyrophosphate; HMG-CoA = hydroxymethylglutaryl CoA; IPP = isopentenyl diphosphate; M5P = mevalonate-5-phosphate; MVA = mevalonate; MVAPP = mevalonate pyrophosphate.

Engineering biology routes to (*R*)-linalool using the MVA precursor upregulation pathway have been described for *E. coli* (Leferink et al., 2016; Karuppiah et al., 2017; Wang et al., 2019; Ferraz et al., 2021; Wu et al., 2021) and more recently other organisms (Nitta et al., 2021; Zhang et al., 2021) (Figure 1). As linalool is cytotoxic to microorganisms (Liu et al., 2020), microbial production can suffer from the instability of DNA pathway constructs and wide variability in production titers. Therefore, mitigations are needed to ensure stable maintenance of a fully functioning metabolic pathway by minimizing genetic recombination that leads to enzyme(s)/pathway loss. Increasing the tolerance of the microbial host to linalool could also be explored to reduce selection of mutated strains that lack a fully functional pathway during fermentation.

Techno-economic analyses of terpenoid production indicates that the fermentation feedstock is a major cost component (Wu and Maravelias, 2018). Feedstock cost and availability are important factors in determining the cost-competitive basis of bioproduction processes. Techno-economic analysis for the bioproduction of the related acyclic monoterpenoid limonene identified that titers and product monetary value impact significantly on the commercial viability of conceptual production processes (Sun et al., 2020). Studies of the scaled biomanufacture of linalool and other monoterpenoids are therefore needed to increase and stabilize production titers, whilst simultaneously shifting production towards the use of widely available and inexpensive carbon feedstocks. The most abundant low-cost renewable and sustainable biomass available is lignocellulose waste (Isikgor and Becer, 2015), such as crop residue (Saini et al., 2015) and paper mill wastewater secondary paper fibers (Suvachan et al., 2021). However, lignocellulose waste requires mechanical and thermochemical pre-processing to release the cellulose fibers, followed by cost-prohibitive enzymatic saccharification by commercial cellulases to release glucose as a carbon source for microorganisms. By contrast, waste secondary paper fibers derived from paper mill industries have already undergone pre-processing, which reduces the overall cost burden of using this biomass as a carbon feedstock. Pulp and paper industries currently produce 400 MT paper per annum and are under intense pressure to make production processes greener by valorizing effluent streams. An attractive opportunity would be to utilize secondary paper fibers as an abundant high-density carbon source for industrial chemicals production. With this in mind, we took a broad-based, multi-targeted approach to identify important factors that would enable the use of relatively low-cost media with a stable *E. coli* host engineered to produce linalool. Specifically, we investigated the efficacy of using engineering biology strategies that were designed to a) increase genetic construct stability; b) enhance host tolerance to linalool; c) determine the effects of genomic integration on linalool production titers; d) explore the influence of production pathway copy number on linalool titer; e) discover the impact on bioproduction of switching to low-cost feedstocks, comprising minimal media and Indian paper mill wastewater secondary paper fibers as sole carbon source.

## Materials and Methods

### Chemicals and Media

Reagents used in this work were commercially sourced and were of analytical grade or better. Industrially-sourced waste paper fines (JK Paper Ltd, India) were kindly supplied by Dr Binod Parameswaran (CSIR NIIST, India) (Suvachan et al., 2021). Culture media pre-blended preparations were obtained from Formedium. The standard *E. coli* DH5α culture medium used for cloning was Luria-Bertani (LB), which is composed of 10 g/L tryptone, 5 g/L yeast extract, and 5 g/L NaCl. For linalool production assays the culture medium was Terrific Broth (TB), containing 24 g/L yeast extract, 12 g/L tryptone, 9.4 g/L KH_2_PO_4_ and 2.2 g/L K_2_HPO_4_. When antibiotic selection was required, the culture medium was supplemented with carbenicillin (50 μg/ml), hygromycin (150 μg/ml), kanamycin (50 μg/ml for plasmids or 30 μg/ml for integrated constructs) or chloramphenicol (34 μg/ml for plasmids or 10 μg/ml for integrated constructs). Agar plates were composed of LB containing 1.5% (w/v) agarose and the required antibiotic (Table 1). Growth of *E. coli* DH5α on mineral based medium with cellulose-based carbon sources was performed using a thiamine supplemented medium described previously (Li and Sha, 2017). For *E. coli* BL21 strain, growth on paper as a carbon source was performed using M9 minimal salts with modified trace element supplementation (3 g/L KH_2_PO_4_, 0.5 g/L NaCl, 6.78 g/L Na_2_HPO_4_, 1 g/L H_4_Cl, 1 mM MgSO_4_, 0.3 mM CaCl_2_, 1 mg/L thiamine, 1 mg/L biotin, 17 µM FeCl_3_, 84 mg/L EDTA, 150 mg/L MnCl_2_.4H_2_O, 105 mg/L ZnSO_4_.7H_2_O, 30 mg/L H_3_BO_3_, 25 mg/L Na_2_MoO_4_.2H_2_O, 15 mg/L CuCl_2_.2H_2_O) (Blank et al., 2008).

**TABLE 1:** Linalool pathway plasmids and genomic integrated strains used in this study.

Construct	Description^1^	Source/Reference
Plasmid constructs		
pMVA	p15A, Kan^R^, *P*_*lacUV5*_-MevT-T_rrnBT1_-T_T7TE_, *P*_*trc*_-MevB-T_rrnBT1_-T_T7TE_	Leferink et al. (2016)
pGLinS	pBBR1, Amp^R^, *P*_*Tet*_-GLinS-T_rrnBT1_-T_T7TE_	Karuppiah et al. (2017)
pMVA-GLinS	p15A, Amp^R^, *P*_*lacUV5*_-MevT-T_rrnBT1_-T_T7TE_, *P*_*trc*_-MevB, *P*_*trc*_-GLinS-T_rrnBT1_-T_T7TE_	(Karuppiah et al., 2017; Ferraz et al. (2021)
	
pMVA-GLinS NR1	p15A, Amp^R^, *P*_*lacUV5*_-MevT, *P*_*trc*_-MevB, *P*_*trc*_-GLinS-T_rrnBT1_-T_T7TE_	This work
pMVA-GLinS NR2	p15A, Amp^R^, *P*_*lacUV5*_-MevT, *P*_*J23116*_-MevB, *P*_*trc*_-GLinS-T_rrnBT1_-T_T7TE_	This work
pMVA-GLinS_L72M_ NR2	p15A, Amp^R^, *P*_*lacUV5*_-MevT, *P*_*J23116*_-MevB, *P*_*trc*_-GLinS L72M-T_rrnBT1_-T_T7TE_	This work
pMVA-GLinS_L72M/V214I_ NR2	p15A, Amp^R^, *P*_*lacUV5*_-MevT, *P*_*J23116*_-MevB, *P*_*trc*_-GLinS L72M V214I-T_rrnBT1_-T_T7TE_	This work
pMVA-G-CmR29*LinS NR2	p15A, Amp^R^, *P*_*lacUV5*_-MevT, *P*_*J23116*_-MevB, *P*_*trc*_-G-CmR29*LinS-T_rrnBT1_-T_T7TE_	This work^2^
pMVA-NLinS NR2	p15A, Amp^R^, *P*_*lacUV5*_-MevT, *P*_*J23116*_-MevB, *P*_*trc*_-NLinS-T_rrnBT1_-T_T7TE_	This work
pMVA-NGLinS NR2	p15A, Amp^R^, *P*_*lacUV5*_-MevT, *P*_*J23116*_-MevB, *P*_*trc*_-NGLinS-T_rrnBT1_-T_T7TE_	This work
Genome integrated *E. coli* strains		
GL	DH5α *arsB*::*P*_*Tet*_-GLinS	This work
M1	DH5α *lacZ*::*P*_*trc*_-MevB, *rbsAR*::*P*_*lacUV5*_-MevT	This work
MGL	DH5α *lacZ*::*P*_*trc*_-MevB, *rbsAR*::*P*_*lacUV5*_-MevT, *arsB*::*P*_*Tet*_-GLinS	This work

1 For plasmids: replication origin, antibiotic marker and promoter-operon-terminator; for genome integrated constructs: *E. coli* strain, loci and promoter-operon.

2 Plasmid pMVA-G-CmR29*LinS NR2 was kindly supplied by Dr Robin Hoeven (University of Manchester, United Kingdom). Plasmids used as templates for the generation of these constructs are described in Supplementary Table S1.

### Plasmid Constructs and Engineering

*E. coli* genomic DNA was extracted and purified using the DNeasy Blood & Tissue Kits (Qiagen). CloneAmp HiFi PCR Premix (Takara Bio) was used for the construction of plasmids, while diagnostic PCR and sample preparation for sequencing was performed using GoTaq Green Master Mix (Promega). Each PCR product was analyzed by agarose gel electrophoresis, followed by purification with NucleoSpin Gel and PCR Clean-up kit (Macherey-Nagel). Plasmid purification was performed using the ISOLATE II Plasmid Mini Kit (Bioline). DNA concentration was measured using a NanoDrop 2000 (Cole-Palmer). The Q5 Site-Directed Mutagenesis Kit was obtained from New England BioLabs. Restriction enzymes for plasmid construction were purchased from New England BioLabs. All other plasmid recircularization and ligations were performed using the In-Fusion cloning kit (Takara Bio).

Gene sequencing and oligonucleotide synthesis were performed by Eurofins MWG (Ebersberg, Germany). To facilitate plasmid construction, DNA primers were designed to contain either 5’ and 3’ restriction endonuclease sites or 15–30 bp overlaps homologous to the target construct for ligation by In-Fusion cloning. The list of primers used for PCR reactions are shown in Supplementary Table S1. Details of the biocatalytic plasmid and chromosomal constructs used in this study are described in Table 1. Plasmids acting as templates for the assembly of linalool production pathway constructs and to facilitate genomic integration of DNA are described in Supplementary Table S2.

After construct assembly, each plasmid was transformed into *E. coli* strain DH5α and incubated overnight on antibiotic-selective LB agar plates. Individual colonies were picked, and small liquid cultures (5 ml) were cultivated overnight at 37°C using antibiotic-selective LB medium (Table 1). Following plasmid recovery and purification, each plasmid was sequenced to confirm the correct construct had been assembled. At each stage of cloning the protocols specified by the individual kit manufacturers were followed.

Plasmid pMVA is a derivative of the limonene-producing construct pJBEI6410 (Alonso-Gutierrez et al., 2013) lacking the genes geranyl pyrophosphate synthase (GPPS) and limonene synthase (Leferink et al., 2016). It contains operons for the upper and lower MVA pathway (MevT and MevB, respectively), controlled by the *lac*UV5 and *trc* promoters, respectively (Figure 1). Construct pGLinS contains the *Abies grandis* geranyl pyrophosphate synthase (AgGPPS) and *Streptomyces clavuligerus* linalool synthase (LinS) controlled by a *tet* promoter (Karuppiah et al., 2017). Plasmid pMVA-GLinS is a combination of pMVA and pGLinS (Figure 1), with the latter operon controlled by a *trc* promoter (Ferraz et al., 2021).

**FIGURE 2:**
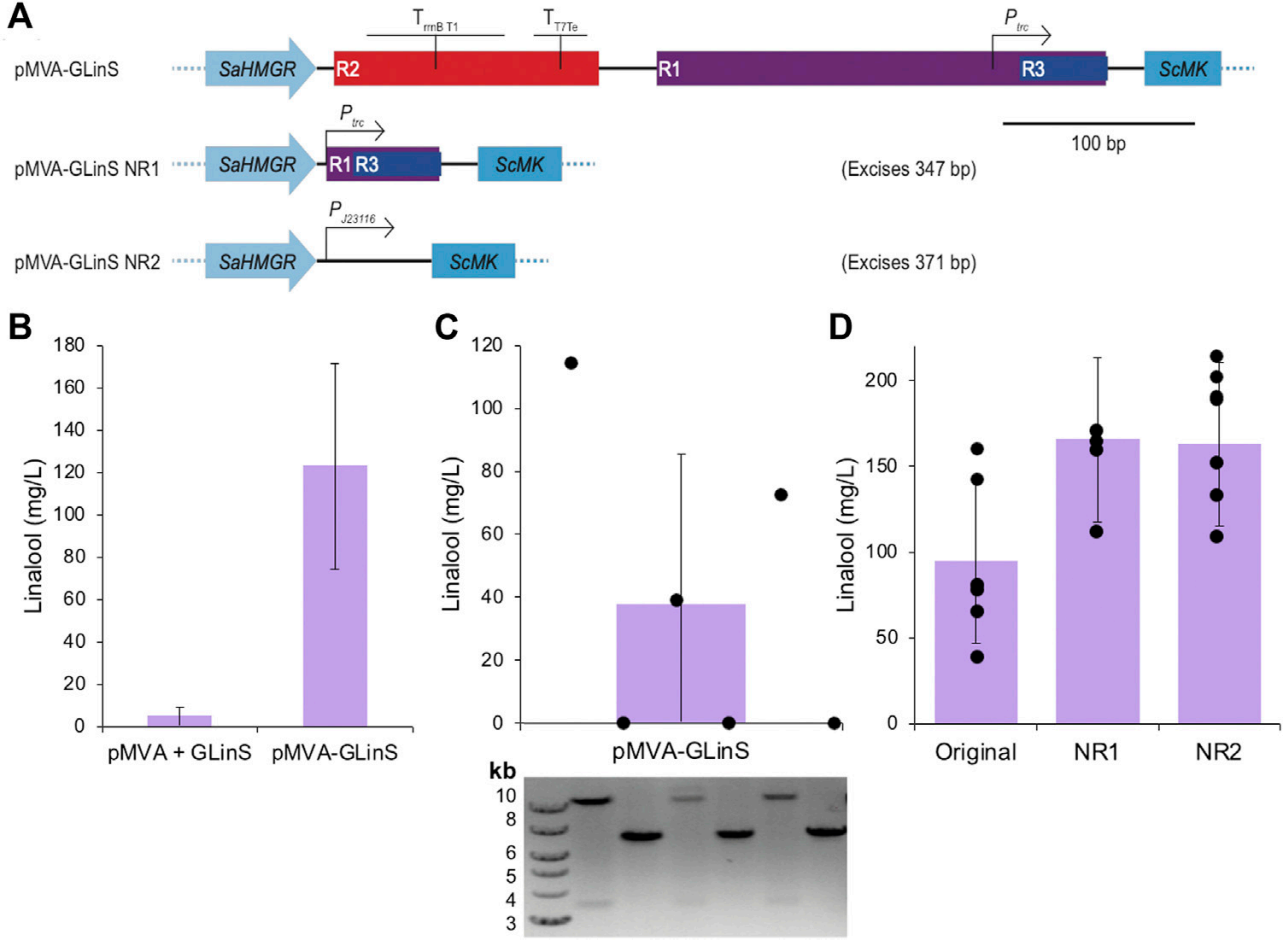
Linalool production by recombinant plasmid-based *E. coli* DH5α. **(A)** Schematic of the method of elimination of repeating regions R1, R2, and R3 of pMVA-GLinS and incorporation of the constitutive promoter *P*_J23116_ to control the expression of the MevB lower MVA pathway enzymes. **(B–C)** Linalool production of biological replicates of *E. coli* DH5α containing the one or two plasmid system. Cultures (3 ml) were incubated in TB medium containing 0.4% glucose and antibiotic selection at 37°C until growth was visible, followed by induction with 50 µM IPTG. A further incubation at 30°C was performed for 68–72 h and linalool production was determined by GCMS analysis. Part C also contains a diagnostic agarose gel electrophoresis of the plasmid fragmentation pattern of the biological replicates suggestive of potential recombination events. The plasmid diagnostic gel lanes are aligned vertically with the linalool data points (black circles), which are individual linalool assays of single colonies of *E. coli* DH5α containing pMVA-GLinS. Possible recombination events are detailed in Supplementary Figure S2. **(D)** Variation in biological replicates in linalool assays by *E. coli* DH5α containing the original pMVA-GLinS and variants NR1-NR2. Error bars represent one standard deviation of the data. Individual biological replicates are shown as black spheres.

Elimination of the repeated regions R1-R3 (see below) in pMVA-GLinS was performed in two stages. The initial phase involved amplifying the whole plasmid by PCR, which selectively deleted region R2 and the majority of region R1 (Figure 2A). This was followed by plasmid recirculation and ligation by In-Fusion cloning to generate pMVA-GLinS NR1. A second round of whole plasmid PCR amplification was performed that eliminated R2 and the residual 3’ end of R1. In addition, the MevB pathway *trc* promoter was substituted for the constitutive promoter *P*_*J23116*_. This PCR product was ligated by In-Fusion cloning to generate pMVA-GLinS NR2.

A variety of modifications of the base pMVA-GLinS NR2 plasmid were performed to increase the expression and/or functioning of the terminal pathway genes *gpps* and/or *linS*. A modified version of pMVA-GLinS NR2 contains a N-terminal 29 amino acid solubility tag from the chloramphenicol resistance marker on LinS (pMVA-G-CmR29*LinS NR2). This plasmid was kindly supplied by Dr Robin Hoeven (University of Manchester, United Kingdom). Whole plasmid PCR amplification was performed on pMVA-GLinS NR2 to substitute wild-type LinS with either a single (L72M) or double (L72M/V214I) variant from pET-bLinS_L72M_ or pET-bLinS_L72M/V214I_ plasmids, respectively (Ferraz et al., 2021). New pMVA-GLinS NR2 constructs incorporating variant LinS genes were assembled by In-Fusion cloning (Table 1). The pMVA-GLinS NR2 plasmid also underwent modification of the LinS ribosomal binding site (aat---GGA​GCT​TTT​TAG​AAG​GAG​GTA​TAG), which also included the removal of the upstream stop codon to generate a GPPS-LinS fusion protein. Finally, pMVA-GLinS NR2 underwent the insertion of neryl pyrophosphate synthase (NPPS) from *Solanum lycopersicum* (GenBank: NM_001,247,704.1) with or without *gpps* gene deletion (Table 1). Further details of the latter two plasmid modification strategies can be found in the Supplementary Methods document.

### Strain Engineering Through Chromosomal Integration

Commercially available chemically competent *E. coli* strains DH5α or BL21 (New England BioLabs) were used for routine cloning of plasmids. The *E. coli* PIR2 strain (Invitrogen) was used for cloning and maintenance of pKIKO plasmids (Supplementary Table S1). This strain harbors the *λpir* gene that encodes the π protein to support the R6K replication origin in pKIKO plasmids (Sabri et al., 2013). DNA transfer into *E. coli* was performed by transformation of chemically competent cells (plasmid construction) or electroporation (construction of genome integrated strains), the latter of which is described in Supplementary Methods. Chromosomal integration pKIKO plasmids were obtained from AddGene (Sabri et al., 2013). These plasmids contain homology arms that allow for integration at the *lacZ*, *arsB*, and *rbsAR* genes in *E. coli* (Sabri et al., 2013). An additional set of plasmids (pKIKO*gltA*Cm and pKIKO*gltA*Km) were generated incorporating a fourth loci (*gltA*) for chromosomal insertion, as described in the Supplementary Methods **(** Supplementary Figure S1). Other plasmids utilized for pKIKO-based chromosomal integration of DNA constructs were pSIM18 (Chan et al., 2007) and pCP20 (Cherepanov and Wackernagel, 1995) (Supplementary Table S1). pSIM18 is a heat-sensitive plasmid that harbors the λ-RED recombination gene (Datta et al., 2006). The recombinase flippase system for antibiotic resistance excision from the genome is encoded on the heat sensitive plasmid pCP20 (Cherepanov and Wackernagel, 1995; Datsenko and Wanner, 2000).

The methods used for chromosomal integration are based on the λ-RED recombination system (Thomason et al., 2014) using the pKIKO vector series of plasmids (Supplementary Table S1) (Sabri et al., 2013; Jervis et al., 2021) as described previously. Further details are provided in the Supplementary Methods. In this general protocol, DNA cassettes for integration were inserted upstream of the kanamycin or chloramphenicol resistance gene and between the specific chromosomal loci homology arms (HA1 and HA2). The antibiotic resistance gene was enclosed on each side by the flippase-mediated recombination sequence (FRT) (Sabri et al., 2013). The linear DNA cassettes used for each genomic integration (HA1-pathway-HA2-FRT-antibiotic gene-FRT) was generated by PCR amplification and electroporated into pSIM18 plasmid-containing *E. coli* DH5α. Confirmation of the presence of the integrated DNA cassette was performed by PCR, using primers specific to regions outside of the chromosome HA1 and HA2 sites, or with one primer specific for the inserted DNA. Following curing of the heat-sensitive pSIM18 plasmid, elimination of the integrated antibiotic resistance gene flanked by FRT sites was performed using the recombinase flippase system encoded on the heat sensitive plasmid pCP20 (Cherepanov and Wackernagel, 1995; Datsenko and Wanner, 2000). Final confirmation of successful gene cassette integration and antibiotic removal was performed by sequencing the genomic DNA in the region of interest.

To integrate the GLinS cassette into the *arsB* loci in *E. coli* DH5α, we constructed the plasmid pKIKO*arsB*Cm:GLinS using PCR and restriction enzyme cloning (Table 1). The GPPS and LinS genes and regulatory elements were PCR amplified from pGLinS and digested with enzymes SacI and SalI. This DNA cassette was ligated to pKIKO*arsB*Cm plasmid previously digested with the same restriction enzymes. Following integration into the chromosome of *E. coli* DH5α, the new strain (GL) was transformed with the pMVA plasmid to supply the geranyl pyrophosphate precursor for linalool production.

To incorporate the MVA pathway into *E. coli* DH5α or the GL strain, the pathway was divided into the upper MevT and lower MevB operons (Figure 1) (Alonso-Gutierrez et al., 2013) for integration at loci *rbsAR* and *lacZ*, respectively. The upper pathway integration plasmid pKIKO*rbsAR*Cm:MevT was assembled using the PCR and restriction enzyme cloning protocol described above. For the lower MevB pathway, the integration plasmid pKIKO*lacZ*Cm:MevB was assembled by PCR amplification of the three-gene operon with its regulatory elements, PCR linearization of pKIKO*lacZ*Cm and ligation by In-Fusion cloning. Following successful genomic integration, the MVA pathway integrated strain (M1) was transformed with pGLinS to allow linalool production to occur. The strain containing the MevT, MevB, and GLinS cassettes (MGL) did not require any plasmid addition.

### Linalool Production

The general protocol for *in vivo* linalool production in *E. coli* was performed using individual colonies from freshly transformed cultures (plasmid constructs only) or overnight liquid cultures as the starting inoculum. Cultures (3 ml) were incubated in TB medium containing 0.4% glucose in sealed glass bottles and incubated at 37°C and 200 rpm until growth became visible (up to 7 h). Recombinant protein expression was induced with 50 µM isopropyl β-D-1-thiogalactopyranoside (*trc* and *lacUV5* promoters) and in some cases with 25 nM anhydrotetracycline (*tet* promoter; Table 1). Cultures were incubated for a further 68–72 h at 30°C. In some assays, 300–600 μL of nonane or isopropyl myristate (organic overlay) was added to the cultures after induction to sequester linalool from the culture medium. Linalool production from cellulose was performed as above (no overlay), except growth was performed in M9 medium containing 1.5% carbon source (glucose or cellulose) with DH5α or 0.5% carbon source (glucose or cellulose) with BL21 (DE3) and 1% Cellic cTec2 cellulase enzyme blend (Sigma-Aldrich).

Linalool titers were determined by extracting 1 ml of culture or organic overlay with an equal volume of ethyl acetate containing 0.01% (v/v) *sec*-butyl benzene (internal standard). The organic layer was dried with anhydrous MgSO_4_ and analyzed for linalool content by GCMS. To assess the retention of the recombinant plasmids (where applicable), minipreps were performed on 1–2 ml of each culture. Each purified plasmid underwent a diagnostic restriction digest with EcoRI and XhoI, and the fragments were analyzed by electrophoresis using a 0.5% agarose gel. The predicted fragment DNA band sizes and plasmid maps for pMVA-GLinS and potential recombined constructs are shown in Supplementary Figure S2.

### Linalool Toxicity and Adaptive Laboratory Evolution

Linalool toxicity studies were performed by monitoring the optical density of cultures exposed to linalool. The starter culture of *E. coli* DH5α was incubated overnight in LB medium (5 ml) with agitation. Replicate aliquots of *E. coli* DH5α (200 μL; 2% inoculum) were set up in LB medium containing 0–2 g/L linalool in a 96-well microtiter plate (Costar). Cultures were covered with a Moisture Barrier Seal (4titude) and incubated at 30°C with 500 rpm agitation within a FLUOstar Omega Microplate reader (BMG Labtech). Optical density readings (OD_600 nm_) were performed every 15 min for 24 h.

Adaptive laboratory evolution (ALE) of *E. coli* DH5α for increased linalool tolerance was performed by exposure of cultures to increasing concentrations of linalool. The initial starter culture was composed of *E. coli* DH5α in 5 ml LB containing 0.5 g/L linalool, which was cultivated overnight at 30°C and 200 rpm. This culture served as the inoculum for subsequent cultures of increasing linalool concentrations (2–20 g/L). Growth was monitored in a microtiter plate reader, as described above. This was performed iteratively until growth was observed up to 20 g/L linalool, with the new strains designated as *E. coli* ALE.

### Saccharification of Cellulose Samples

Paper fines from a waste stream of JK Paper Ltd paper mill were used as a low-cost cellulose source. The paper was sanitized by vortexing in 100% ethanol, followed by solvent evaporation in a sterile environment. To determine the effective glucose titers from paper fines, saccharification was performed using the Novozymes Cellic cTec2 cellulase enzyme blend (Sigma-Aldrich) using an adaptation of the NREL procedure (Resch et al., 2015). Paper fines (100 mg) were suspended in 10 ml of sterile 0.1 M sodium citrate buffer pH 4.8 containing 80 μg/ml tetracycline hydrochloride, 0.002% sodium azide, and 1:100 Novozymes Cellic cTec2 cellulase enzyme blend. Samples were incubated at 50°C with shaking at 250 rpm. A negative control (no paper) was performed to determine the background glucose content present in the Cellic cTec2 cellulase blend. Suspensions were clarified by centrifugation at 3,200 g for 10 min or 17,000 g for 2 min. The aqueous solution was analyzed for glucose content by HPLC.

### Growth on Cellulose as a Carbon Source

Growth of *E. coli* strains DH5α and BL21 (DE3) on cellulose-based carbon sources was performed by cultivation in mineral-based medium containing 0.5% of either glucose (control), carboxymethylcellulose (CMC), Sigmacell cellulose or pretreated paper fines with a 1:1000 inoculum of an overnight culture in LB medium. Simultaneous saccharification and fermentation was performed where cellulose digestion to glucose occurred by the addition of the Cellic cTec2 cellulase blend (1:100) or expression of the Addgene ampicillin-resistant pCellulose multicellulase plasmid (Bokinsky et al., 2011). Recombinant cellulase production was controlled by the *cspD* and *wrbA* promoters that are induced in stationary phase (when *E. coli* is starved for carbon) (Bokinsky et al., 2011). To minimize the likelihood of microbial contamination in cultures containing cTec2, the *E. coli* strain contained the pBbS5a:RFP vector and each culture was cultivated for 24 h at 37°C in the presence of 50 μg/ml carbenicillin. Culture optical density measurements were not possible due to the opaqueness of some of the carbon sources. Therefore, after 24 h of growth, culture aliquots were diluted and spread on LB agar plates and incubated overnight at 37°C. Growth is expressed as the number of colony-forming units (CFUs).

### Analytical Procedures

Linalool production was analyzed by GCMS using an Agilent Technologies 7890B GC equipped with a 5977A MSD detector. Product(s) (1 μL) were separated on a DB-WAX column (30 m × 0.32 mm i.d., 0.25 µM film thickness, Agilent Technologies) using the running conditions described previously (Leferink et al., 2016). Compound identification was carried out using the reference spectra in the NIST library of MS spectra and fragmentation patterns. Linalool quantitation was performed by comparing peak areas to a standard curve generated from authentic standards run under the same conditions. Linalool concentrations obtained from organic overlay samples have been corrected to express the data as mg/L culture.

Glucose release from cellulose was analyzed by HPLC using an Agilent 1260 Infinity HPLC with a 1260 ALS autosampler, TCC SL column heater, a 1260 refractive index detector (RID). Samples were run on an Agilent Hi-Plex Ca column (300 × 7.7 mm) using 100% HPLC-grade water as the mobile phase (0.6 ml/min) at 85°C for 30 min. Glucose quantitation was performed by comparing peak areas to a standard curve generated from authentic standards (retention time of 13 min). For generalized glucose and organic acid detection from cellulose-containing samples, culture supernatant aliquots (20 μL) were injected into an Agilent Hi-Plex H column (7.7 × 300 mm, 8 μm) using 0.005 M H_2_SO_4_ as the mobile phase (0.7 ml/min). The run was performed at 60°C for 60 min with the RI detector set to 55°C. Retention times for each component were: 10 min for glucose, 14 min for formate, 16 min for acetate, 17 min for levulinic acid, 33 min for 5-hydroxymethylfurfural, and 49 min for furfural. Analyte concentrations were calculated by comparing the peak areas to a standard curve generated from analytical standards of known concentrations.

## Results and Discussion

### Plasmid Engineering for Increased Genetic Stability

Bacterial linalool production in *E. coli* has been previously demonstrated using a one or two plasmid-based system incorporating the entire recombinant pathway from acetyl-CoA (Leferink et al., 2016; Karuppiah et al., 2017; Wang et al., 2019; Karuppiah et al., 2020; Wu et al., 2021) or via a non-canonical isopentenol utilization pathway for GPP precursor production (Ferraz et al., 2021). A screen of multiple *E. coli* strains showed that the highest linalool titers were obtained using the K-12 strain DH5α with the two-plasmid recombinant linalool production system (pMVA + pGLinS) (Leferink et al., 2016). This strain is also known to produce higher beta-carotene levels than *E. coli* BL21, MG1655, DH5α, S17-1 and XL1-Blue strains (Yoon et al., 2009). Therefore, we selected *E. coli* DH5α as our base strain for linalool production and used the one plasmid system (pMVA-GLinS; Table 1) as the template construct for our optimization studies (Figure 2A).

In our hands, we found that biological replicates (individual colonies) of *E. coli* DH5α containing either the one or two plasmid system generated linalool titers with a high degree of variability (122.7 ± 48.6 and 4.90 ± 4.5 mg/L, respectively; Figures 2B,C). This suggests the plasmid-based linalool production system is not stably maintained. An apparent correlation was observed where the linalool titer was inversely proportional to the size of the freshly transformed *E. coli* pMVA-GLinS colony used to inoculate the assay medium. This suggested linalool production from leaky promoters adversely affected culture growth, and small colony size was subsequently used as a criterion for selection of higher linalool producers.

Prior studies have suggested that instability in titers of terpenoids and other natural products may be due to plasmid recombination events, which in turn reduces *in vivo* production of toxic intermediates and products generated by full length constructs (Rinaldi et al., 2021). This may be especially relevant for pathways where either large or multiple plasmids need to be maintained. To check for the presence of plasmid recombination events in the linalool production plasmid, diagnostic restriction digestion and agarose gel electrophoresis of replicate cultures were therefore performed. Cultures displaying significant linalool production showed the “full-length” pMVA-GLinS plasmid restriction map (10.9 + 3.7 kb fragments), whereas cultures displaying low, or no titers had different banding patterns (Figure 2C and Supplementary Figure S2). This suggests that pMVA-GLinS is undergoing recombination, which decreases or eliminates the ability of *E. coli* to make linalool.

Studies have shown that repeated sequences as short as 8 bp can recombine both within and between plasmids (Sleight et al., 2010). As DH5α is a Δ*recA E. coli* strain, any recombinant events must be RecA-independent. This can frequently occur in the presence of pairs of 25 bp homology regions, with recombination frequency increasing up to 411 bp (Lovett et al., 2002). A closer examination of pMVA-GLinS revealed the presence of four sets of identical repeating sequences longer than 15 bp, which were a consequence of maintaining multiple, identical regulatory elements for gene expression (Figure 2A and Supplementary Figure S2). Repeated region 1 (R1; 234 bp) contained the *trc* promoter upstream of the lower MVA pathway and GLinS. The second region (R2; 138 bp) contained the *rrnBT1* and *T7Te* transcriptional terminators downstream of GLinS. Repeated region 3 (R3; 46 bp) consisted of the common sequence between the *trc* and *lac*UV5 promoters upstream of each part of the pathway (embedded in R1), thus appearing three times in the plasmid. The final repeating region (R4; 26 bp) corresponded to an RBS upstream of GPPS and LinS. Simulations of potential recombination events are shown in Supplementary Figure S2, which predicts the diagnostic banding patterns seen in some of the non-linalool producing colonies (Figure 2C).

To reduce the likelihood of plasmid recombination, we eliminated regions R1-R3 in two stages to generate pMVA-GLinS variants NR1 and NR2, respectively (Figure 2A). The final pMVA-GLinS NR2 plasmid contained a *P*_J23116_ constitutive promoter in place of *P*_*trc*_ upstream of the lower MevB pathway. This new promoter was selected due to a prediction by SelProm (Jervis et al., 2019) that its subsequent expression levels would be similar to that of the original *trc* promoter. Comparative assays showed that both NR1 and NR2 constructs showed minor (1.7-fold) increases in linalool production compared to the original pMVA-GLinS in *E. coli* (162.8 ± 37.1 for NR2; Figure 2D). While some variability in titres still exists, there was a decrease in the number of non-linalool producing replicates. Other factors that could account for increased titers include the loss of some of the redundant terminator regions and/or the switch of the lower MevB pathway promoter.

It is interesting to note that these repeated regions are derived from the pJBEI-6410 or pJBEI-6409 MVA pathway backbone plasmid (Alonso-Gutierrez et al., 2013) due to the presence of multiple transcriptional control elements with sequence similarities. Variants of these plasmids have been used for the production of multiple terpenoids (Leferink et al., 2016), such as pinene (Bao et al., 2019), geraniol/geranyl acetate (Chacón et al., 2019; Wang et al., 2021), limonene (Rolf et al., 2020), perillyl alcohol (Alonso-Gutierrez et al., 2013) and other linalool production studies (Wu et al., 2021). Other natural product biosynthesis plasmids are known to contain significant repeating regions, such as pBbA5c-MevT (CO)-T1-MBIS(CO, ispA). This plasmid was designed to produce amorphadiene, a precursor of the antimalarial drug artemisinin (Redding-Johanson et al., 2011), and the sesquiterpene α-humulene (Alemdar et al., 2017). Terpenoid production has also been described using constructs based on the pACYCDuet backbone, which has a 51 bp repeated T7 promoter and lac operator (Meng et al., 2011; Liu et al., 2019). In spite of this, only one of these reports describes variable production titers (pinene) due to recombination of a pJBEI-6409-derived plasmid (Bao et al., 2019). In this case, pinene titers were improved by the elimination of repeated regions within the construct.

We hoped to build on the success of titer increase and plasmid stability improvements by implementing additional strategies to increase plasmid retention. One approach was to apply environmental stress by co-expressing with a second plasmid (pBbB2k-RFP) containing a compatible *ori* and a second antibiotic resistance gene (Gama et al., 2020) (Supplementary Figure S3). An alternative approach is to decrease the cytotoxicity of linalool by sequestering it into a microbial compatible organic co-solvent overlay (nonane or isopropyl myristate; Supplementary Figure S4). Monoterpenoid sequestering using this approach has been shown previously (Leferink et al., 2016). We also targeted key bottleneck enzymes GPPS and LinS for modification by a) incorporation of the more active LinS_L72M/V214I_ variant (Ferraz et al., 2021); b) increasing the solubility of LinS by incorporating an N-terminal 29 amino acid tag from the chloramphenicol acetyltransferase enzyme (Wang et al., 2019) c) generation of a GPPS-LinS fusion gene with a small linker region and d) substitution or inclusion of the N-terminally truncated neryl pyrophosphate synthase (NPPS) from tomato (*Solanum lycopersicum*) (Wu et al., 2019) (Supplementary Figure S5). Overall, these approaches showed little to no additional positive effects on linalool production titers and/or plasmid stability. This highlights that strategies shown to impart positive outcomes with one recombinant pathway will not necessarily translate into another. Further discussion on the environmental stress and GPPS/LinS modification approaches related to linalool titres can be found in the Supplementary Material document.

### Enhancing Linalool Tolerance by Laboratory Evolution

Linalool is known to be cytotoxic to microorganisms (Liu et al., 2020). We conjectured this may contribute towards linalool-producing plasmid instability and subsequent titer variability in *E. coli*. We investigated the cytotoxicity of linalool towards *E. coli* DH5α by monitoring its growth profile in the presence of externally supplied linalool. The results indicated that linalool significantly affects cell growth rate at levels < 1 g/L, with high concentrations (20 g/L) leading to a rapid loss of cell density after the addition of linalool (Supplementary Figure S6). Therefore, we performed Adaptive Laboratory Evolution (ALE) to increase the tolerance of *E. coli* DH5α towards linalool by performing continuous cultivation in increasing concentrations of linalool (Rinaldi et al., 2021).

**FIGURE 3:**
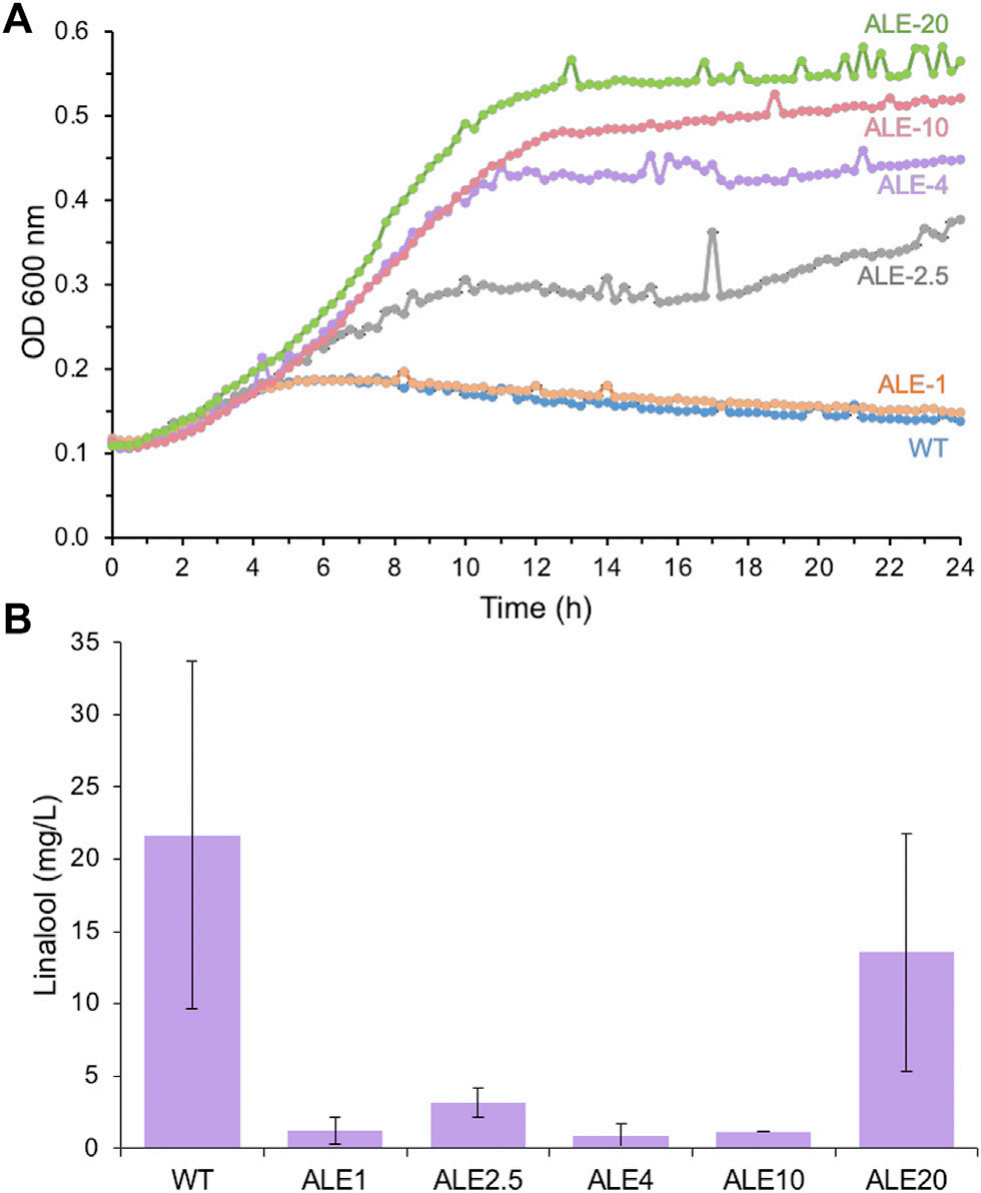
Growth **(A)** and linalool production. **(B)** of wild-type *E. coli* DH5α and adapted laboratory evolution (ALE) strains in the presence of linalool. Replicate aliquots of an overnight *E. coli* DH5α culture (200 μL) were set up in LB medium containing 10 g/L linalool in a sealed 96-well microtiter plate and incubated at 30°C with 500 rpm agitation within a microplate reader. Optical density readings (OD_600 nm_) were performed every 15 min for 24 h. *E. coli* strains: WT (DH5α) = blue; ALE-1 = orange; ALE-2.5 = grey; ALE-4 = purple; ALE-10 = red; ALE-20 = green. Evolved linalool tolerant strains ALE 1, 2.5, 4 10 and 20 were transformed with pMVA-GLinS NR2 and assayed for linalool production as described in Figure 2. Error bars represent one standard deviation of the average of up to 3 individual data points.

The growth profile of a subset of iterative ALE strains in the presence of 0–20 g/L linalool was compared to see if linalool tolerance had improved. Strains ALE-2.5 to 20 showed significant increases in culture growth in the presence of linalool compared to the wild-type strain (Supplementary Figure S6 and Figure 3A). For example, ALE-20 in 5 g/L linalool achieved around 70% of the optical density after 24 h as the same strain without any linalool present. For wild-type DH5α, only ∼ 10% of the growth was achieved in 5 g/L linalool, with a gradual loss of optical density over time. In addition, significant growth was detected in the presence of 10 g/L linalool, a concentration that completely inhibited growth in the wild-type strain (Supplementary Figure S6).

Each of the ALE strains was transformed with the pMVA-GLinS NR2 plasmid to assess whether increasing the linalool tolerance leads to an increase in linalool production titers. Unfortunately, linalool titers of each of the ALE strains were found to be lower than the wild-type strain (Figure 3B). It is not clear why the more tolerant strains do not generate higher titers of linalool, as the mechanism(s) of increased linalool tolerance in the evolved strains has not been determined. A possible explanation is that ALE strains have adapted to decreased uptake of exogenously supplied linalool from the culture medium (lower permeability across the membrane), which could also translate to decreased secretion of intracellular linalool into the culture medium. This could in turn lead to higher accumulation of *in vivo*-generated linalool intracellularly, leading to product feedback inhibition, cytotoxicity or genetic instability of the plasmid. These findings illustrate how trialing an approach shown previously to successfully improve product titers does not necessarily translate into a positive outcome on a different system, even though the same problem (cytoxicity of the product) is present.

### Chromosomal Integration of the Linalool Biosynthetic Pathway

**FIGURE 4:**
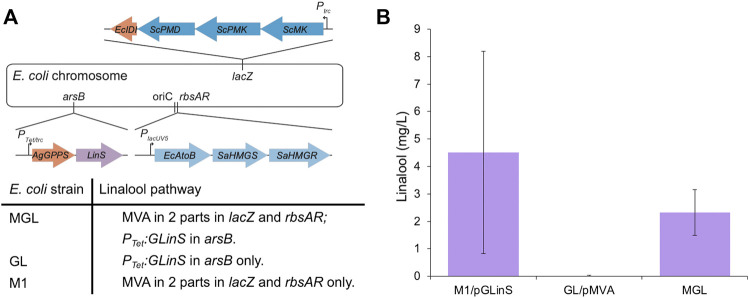
Linalool production in *E. coli* containing genome integrated copies of the pathway. **(A)** Location of the three constitutive parts of the linalool-producing constructs integrated within different recombinant *E. coli* strains. The three loci where DNA cassettes were inserted were *arsB*, *rbsAR* and *lacZ*. For strains with a genomic MVA pathway integration, the genes *EcAtoB*, *SaHMGS* and *SaHMGR* were inserted at the *rbsAR* loci, while the remaining genes (*ScMK*, *ScPMK*, *ScPMD* and *EcIDI*) were inserted at the *lacZ* loci. **(B)** Linalool production of the partial and fully integrated pathway strains of *E. coli* DH5α and the linalool tolerant evolved strain. Cultures (3 ml) were incubated in TB medium containing 0.4% glucose and antibiotic selection at 37°C until growth was visible, followed by induction with 50 µM IPTG (only M1) and 25 nM anhydrotetracycline. A further incubation at 30°C was performed for 68–72 h and linalool production was determined by GCMS analysis.

The chromosomal integration of recombinant pathways into microbial hosts is generally considered preferable to maintaining genes on a plasmid. This is because it eliminates the need for toxic and/or expensive selection agents (antibiotics) for plasmid maintenance (Friehs, 2004), which could prolong fermentation time and maximize product yields (Tyo et al., 2009; Amer et al., 2020a; Amer et al., 2020b). To this end, we performed partial and full integration of the more genetically stable linalool biosynthetic pathway into *E. coli* DH5α (Figure 4A). The simplest genome integrated system tested contained only integrated GPPS and LinS under control of a tetracycline inducible *P*_*tet*_ promoter (strain GL). Precursor geranyl pyrophosphate (GPP) was supplied by the presence of the MVA pathway on a plasmid. Almost no linalool was visible with the genome integrated GL strain compared to the plasmid-borne system (Figure 4A). A dramatic decrease in linalool titers with genome integrated strains is not surprising, given the genes are now present as a single copy in the genome instead of multiple copies on a plasmid (Amer et al., 2020a). However, such a dramatic titer reduction may have been in part due to the additional metabolic burden placed upon *E. coli* by the necessity to provide two antibiotics for linalool production (tetracycline for GLinS induction and kanamycin for pMVA maintenance). Therefore, additional constructs were incorporated that did not utilize a *P*_*tet*_ promoter.

Complete integration of the linalool production pathway was also explored. Due to the length of the MVA pathway, it was incorporated in two parts (MevT and MevB) into the high expression genome loci *rbsAR* and *lacZ*, respectively (strain M1) (Figure 4A). Incorporation of the pGLinS plasmid resulted in linalool titres of 4.51 ± 3.68 mg/L; (Figure 4B). Titres were reduced to 2.32 ± 0.84 mg/L when the full linalool pathway was incorporated within the genome (strain MGL; Figure 4A and Table 2). However, given the variability among biological replicates, these integrated or partially integrated strains overall showed similar linalool titers, with about a 100-fold reduction compared to using the pMVA-GLinS NR2 plasmid. This is not surprising given that the MVA pathway exists as a single copy on the chromosome compared to the high copy number plasmid-borne construct (Martin et al., 2003; Tyo et al., 2009). Similar terpenoid pathway titer reductions have also been reported by others in genome integration studies (Zhao et al., 2013; Alonso-Gutierrez et al., 2018; Wei et al., 2018).

**TABLE 2:** Comparison of published recombinant terpene pathway-based precursor and product titres from plasmid-based and genomic integrated strains.

Chemical	Titers (mg/L)					Additional engineered components	References
	Highest	Plasmid	Integrated-I	Integrated-F	Pathways^1^		
MVA	47000	14600	∼15000	30000	Upper MVA	−	(Tabata and Hashimoto, 2004; Wang et al. (2016)
Linalool	1523	122.72	2.32	2.32	MVA	−	(Wu et al., 2021); This work
Bisabolene	1150	1150	0.138	0.435	MVA	Promoters of MVA pathway and bisabolene biosynthesis genes	(Alonso-Gutierrez et al., 2015; Alonso-Gutierrez et al. (2018)
*ent*-Kaurene	1872	-	623.4^2^	623.4	MEP	Additional MEP pathway copy, 5′UTR of *ent*-kaurene biosynthesis genes, central metabolism	(Wang et al., 2015; Moon et al. (2020)
Tocotrienol	(1425)	(325)	(604)	(1425)	MEP	Promoters and additional copies of MEP pathway genes	Ghanegaonkar et al. (2013)
Lycopene	[(448)]^3^	-	[(0.66)]	1200	MVA	Lower MVA extra copies & lycopene biosynthesis genes (multiple loci)	(Coussement et al., 2017; Wei et al., 2018; Hussain et al. (2021)
β-Carotene	3600	-	3600^2^	3600	MEP & MVA	Regulation of native MEP, β-carotene biosynthesis promoter, central metabolism	Gong et al. (2020)
Astaxanthin	1180	-	15.45^2^	1180	MEP & MVA	Regulation of native MEP, β-carotene biosynthesis promoter, central metabolism, astaxanthin biosynthesis 2nd copy, chaperones	Gong et al. (2020)

1 Engineered pathways.

2 Initial strain making terpenoid already had some previous engineering.

3 For reference, the next highest titer reported in g/L is 3.52 g/L or 50.6 mg/g DCW lycopene (Sun et al., 2014). Titers in single and double parentheses have units of µg/gCDW and mg/g DCW, respectively. Integrated-I = initial integration; Integrated-F = final integration. MVA = mevalonate.

To obtain higher titers of linalool it is preferable that multiple copies of key bottleneck genes be integrated stably within the genome. This approach was taken with lycopene production from chromosomally-integrated pathways, where titers matched and exceeded those of plasmid-borne sequences by increasing chromosomal copy number (Tyo et al., 2009). A rapid approach of achieving this is to use the CreloxP system for high-copy number integration of gene cassettes using growth under increasing antibiotic resistance as the stimulus for multi-copy insertion, as has been applied for the integration of the *phaCAB* genes at the *asnB* loci (50 copies) in *Halomonas* TD01 (Yin et al., 2015). This may prove to be a viable approach for significantly improving linalool titres in a genomic encoded system. In addition, other approaches to increasing linalool titers could include modifying regulatory elements such as promoters and ribosomal binding sites in native and genomic-integrated sequences. This proved successful in some cases to increase titers to match or even surpass those obtained with plasmids (Table 2) (Tyo et al., 2009; Lemuth et al., 2011; Chen et al., 2013; Ghanegaonkar et al., 2013; Li Y. et al., 2015; Ye et al., 2016; Wei et al., 2018). Given that single copy genomic integration of the entire linalool biosynthetic pathway led to a significant decrease in titers, we decided to focus subsequent optimization studies using the enhanced plasmid-based system.

### Saccharification of Paper Mill Waste to Glucose

We have demonstrated linalool production in *E. coli* DH5α *via* both plasmid-borne and genome integrated pathway incorporation. A key constraint in transitioning this “proof-in-principle” bacterial linalool production into a commercially viable venture is the reduction in costs associated with bacterial cultivation. Standard laboratory *E. coli* rich growth medium (TB) is prohibitively expensive on a large scale, and existing pilot scale bacterial cultivations typically rely on a mineral-based medium supplemented with glucose as a carbon source (Ruiz et al., 2013; Li J. et al., 2015). An abundant supply of glucose can be sourced *via* the enzymatic saccharification of waste lignocellulose biomass (de Souza, 2013; Beig et al., 2021). Paper mill wastewater contains secondary or rejected paper fiber, generated by the depuration of bleached pulp (Suvachan et al., 2021). This fiber has a high cellulose content, and enzymatic saccharification of this waste to glucose has been shown to be a suitable low cost carbon source for the growth of lysine-producing *Corynebacterium glutamicum* (Suvachan et al., 2021). Therefore, we investigated the potential of hydrolyzed paper as a feedstock for *E. coli* linalool production.

We tested the ability of a commercial cellulase blend to release glucose from Indian paper mill secondary fiber samples. This includes acid and base pre-treated fibers (Suvachan et al., 2021), designed to improve the access of saccharolytic enzymes to the cellulose (Elliston et al., 2015). Saccharification studies yielded maximal glucose titers [∼ 70% (w/w)] between 24 and 48 h (Supplementary Figure S7). These titers are consistent with compositional analysis of paper fibers showing a more than 50% cellulose content (Aguayo et al., 2018; Suvachan et al., 2021). The acid or base pre-treatment did not appear to have influenced the rate of glucose release or final glucose titers significantly. This is surprising given that previous studies showed acid pre-treatment removes the hemicellulose fraction in the secondary fiber, thereby making enzymatic saccharification more efficient (Suvachan et al., 2021). However, given that excess levels of cellulase blend were added to the saccharification reaction, any significant differences in the glucose release rate within the first 24 h would not be apparent.

An additional consideration for the use of secondary fiber as a bacterial carbon source is the potential for the presence of microbial growth inhibitors generated during the paper making process. However, analytical HPLC of aqueous secondary fiber extracts showed no evidence of significant levels of known growth inhibitors furfural, 5-hydroxymethylfurfural, acetate, formate or levulinic acid (Elliston et al., 2015). Therefore, glucose release from secondary fiber has potential to be a low-cost carbon source for *E. coli* growth.

### Simultaneous Saccharification and Fermentation for Linalool Production

Standard mineral-based growth medium with a single carbon source is not suitable for scaled cultivation of *E. coli* DH5α due to it being a thiamine auxotroph (*thi* ^−^) (Durfee et al., 2008) and the presence of a *purB* mutation (Jung et al., 2010). In contrast, *E. coli* BL21-based strains grow more efficiently in a mineral-based medium without the addition of thiamine. Therefore, we investigated the ability of both DH5α and BL21 strains to generate linalool in cellulose-based carbon sources expressing the pMVA-GLinS NR2 plasmid. We initially looked at growth of *E. coli* BL21 on cellulose-based carbon sources in the absence of pMVA-GLinS NR2 plasmid by indirectly monitoring colony forming units. This was due to the opaqueness of the paper-containing growth medium that prevented growth monitoring by optical density (see Supplementary Results for further details). The direct addition of glucose derived from saccharification of secondary fibers to culture medium was not performed due to the addition of biocides to prevent contamination during cellulolytic degradation. Instead, simultaneous saccharification and fermentation was performed whereby the carbon source was added direct to the minimal medium in addition to the cTec2 cellulase blend or via co-expression of a pCellulose plasmid (Bokinsky et al., 2011). Growth was higher when the cTec2 enzyme blend was added to the culture as the means of releasing glucose from the cellulolytic carbon sources (Supplementary Figure S8). We therefore used this latter approach to screen both *E. coli* strains for linalool production on a variety of cellulose-based substrates.

**FIGURE 5:**
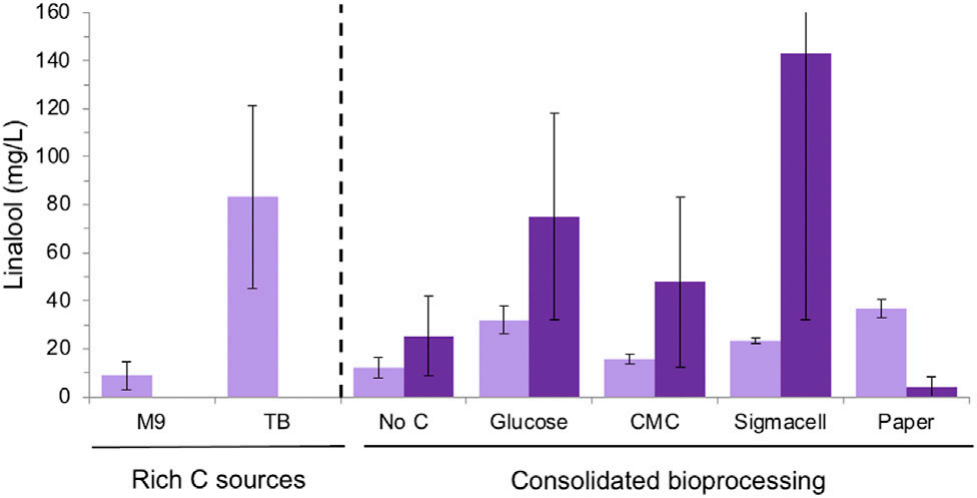
Linalool production of *E. coli* DH5α and BL21 (DE3) strains expressing pMVA-GLinS NR2 on cellulose-based carbon sources. Simultaneous saccharification and fermentation was performed in minimal medium with supplemental thiamine for the DH5α strain. Glucose release from cellulose-based medium was performed *via* the addition of the cTec2 enzyme blend. The error bars represent one standard deviations of a minimum of triplicate assays. *E. coli* strains: DH5α = dark purple; BL21 = light purple.

Overall, *E. coli* strain DH5α generated higher linalool titres compared to BL21 for most carbon sources, although the errors were higher with the former strain (Figure 5). This is consistent with previous observations that linalool titers tend to be the highest for the DH5α strain (Yoon et al., 2009; Han et al., 2016; Leferink et al., 2016). Minor linalool production was detected in control cultures without an added carbon source due to the presence of the commercial cellulase, whose preparations often contain sugar. The highest linalool titres for DH5α were seen with Sigmacell cellulose and glucose as the carbon sources (143 ± 111 and 75 ± 43 mg/L, respectively). In contrast, BL21 cultures generated similar titres of linalool with both glucose (32.03 ± 5.71 mg/L) or wastepaper (36.90 ± 3.79 mg/L). These titers are lower than those obtained in the presence of rich medium (83.3 ± 38.1 mg/L), but higher than for CMC and Sigmacell cellulose carbon sources. Additionally, terpenoid titers tend to be lower when *E. coli* is cultivated in minimal media compared to in rich media (Vickers and Sabri, 2015). This may be due in part to the observation that plasmid loss tended to be higher in minimal medium [data not shown; (Friehs, 2004)], possibly due to rich media relieving metabolic burden and alleviating stress (Singha et al., 2017).

Surprisingly, DH5α cultures showed poor linalool titres on wastepaper fibers compared to other cellulose-based substrates and the BL21 strain cultivated on paper (4.07 ± 4.08 mg/L; Figure 5). In addition, the variability of titres amongst biological replicates was higher for DH5α cultures compared to BL21 when cultivated on minimal medium. The low linalool titres of DH5α cultures grown on paper can be explained by the drastic reduction seen in plasmid levels compared to other carbon sources (Supplementary Figure S9). However, the relative stability of the pMVA-GLinS NR2 plasmid can be seen in non-paper cultures as a significant reduction in the appearance of apparent recombination events, although a few colonies appeared to have lost the plasmid during cultivation. This suggests other factors may be impacting on the high variability in linalool titres beyond plasmid effects, such as differences in the level of fitness of *E. coli* DH5α vs. the BL21 strain in minimal medium. In addition, the volatility of linalool results in losses into the headspace of the assay cultures, which can significantly impact on the recovery after a prolonged incubation.

Despite the complications discussed above, to our knowledge, this is the first demonstration of linalool production from lignocellulose waste, and is encouraging given similar titers are obtained with comparative studies with glucose as the sole carbon source. Previous studies have shown that careful optimization studies from lab scale to 1.3 L bioreactors can lead to significant increases of linalool titers from 100–270 mg/L to 1–1.5 g/L (Wang et al., 2019; Wu et al., 2021). Scaled fermentations under controlled process conditions could impact on the reproducibility of linalool titres by the careful control over carbon source feeding and the use of exhaust gas condensers to improve the retention of linalool in the liquid phase. Therefore, further studies are required beyond this “proof-of-principle” demonstration to improve the scaling potential of the process by optimizing the culture conditions to increase linalool titers and switching from adding commercially sourced cellulases to the efficient co-expression of secretable cellulolytic enzymes (Li et al., 2021) and cytoplasmic monoterpenoid production pathways.

## Conclusions and Future Perspectives

Increasingly in the literature there are studies describing the proof-of-principle demonstration of laboratory scale chemicals, pharmaceuticals, and advanced synthetic fuels production through synthetic biology routes. Yet the transition from laboratory demonstration to generating a viable scaled process remains elusive for most of these potential “green” routes to chemicals production. This is often due to difficulties with product titers, stability of the system and/or the relative costs of scaled fermentation compared to general synthetic routes. This study has endeavored to initiate the transition of microbial linalool production towards a scaled commercially viable venture. Key bottlenecks identified as roadblocks for scaling realization were linalool titers, instability of the genetic constructs, requirements for plasmid maintenance (toxicity and associated costs) and the use of expensive feedstocks.

We have clearly demonstrated that not all traditional routes to improving the robustness of a microbial process and increasing product titres can be applied to every recombinant system. Key to success is identifying the key bottlenecks in the process and designing strategies to circumvent these limitations. For the linalool story, stabilization of genetic constructs proved to be a key step forward in both increasing titres and maintaining full-length plasmid throughout the duration of the fermentation. Further studies are required to optimize multi-copy number integration of key bottleneck genes, eliminate the need for chemical induction and improve the retention of linalool into the liquid phase for improved reproducibility of titres. Given the significant differences in linalool titres among *E. coli* strains, screening of multiple potential strains would be advantageous to find the one that is the most suitable for growth on cellulose-based carbon sources. This will enable the linalool titers to meet or even surpass those seen with plasmid-based systems.

To tackle the high costs of scaled microbial fermentations, we investigated the potential of wastepaper mill secondary fiber as a sole carbon source with inexpensive mineral based medium. This cellulose-rich waste product was shown to be readily converted into glucose at a high yield by the addition of commercial cellulases. However, saccharification of cellulose by commercially sourced cellulases remains the major cost associated with microbial fermentation from lignocellulose waste. Therefore, we demonstrated that simultaneous saccharification and fermentation was possible, generating higher linalool titers than when cultivated on glucose alone as the sole carbon source. In addition, the use of a consolidated bioprocessing approach of co-expression of cellulolytic enzymes also supported growth on cellulose. Further studies could reduce the costs associated with scaled microbial fermentations by improving the expression and/or secretion of cellulases within *E. coli* to maximize glucose release. Alternatively, the addition of an inexpensive self-made cellulase blend extracts sourced from naturally cellulolytic organisms could be a cost-effective and efficient way of performing simultaneous saccharification and fermentation. While further studies are required to improve linalool production overall, this work has identified the importance of isolating genetically stable production strains, whilst demonstrating that the targeting of several other commonly studied characteristics have been less important in improving production titres. The work shows progress towards tipping the balance of microbial linalool production towards scaling viability whilst emphasizing multifactorial issues that need to be considered. In all likelihood, more holistic approaches involving further rounds of microbial engineering, coupled to fermentation process development and product recovery, will be required to enable the development of robust, scaled methods for linalool production from engineered *E. coli* strains.

## Data Availability

The original contributions presented in the study are included in the article/Supplementary Material, further inquiries can be directed to the corresponding author.

## Abbreviations

AcAcCoA, acetoacetyl-CoA; AcCoA, acetyl-CoA; ALE, Adaptive Laboratory Evolution; AgGPPS, *Abies grandis* geranyl pyrophosphate synthase; DMAPP, dimethylallyl diphosphate; EcAtoB, *Escherichia coli* acetyl-CoA acetyltransferase; EcIDI, *E. coli* isopentenyl pyrophosphate isomerase; GPP, geranylpyrophosphate; HMG-CoA, hydroxymethylglutaryl CoA; IPP, isopentenyl diphosphate; IPM, isopropyl myristate; LinS, *Streptomyces clavuligerus* linalool synthase; M5P, mevalonate-5-phosphate; MevB, lower MVA pathway; MevT, upper MVA pathway; MVA, mevalonate; MVAPP, mevalonate pyrophosphate; SaHMGR, *Staphylococcus aureus* 3-hydroxy-3-methylglutaryl-CoA reductase; SaHMGS, *S. aureus* 3-hydroxy-3-methylglutaryl-CoA synthase; ScMK, *Saccharomyces cerevisiae* mevalonate kinase; ScPMD, *S. cerevisiae* phosphomevalonate decarboxylase; ScPMK, *S. cerevisiae* phosphomevalonate kinase.
