# Supplementary material for: Bioproduction of Linalool From Paper Mill Waste
Source: Front Bioeng Biotechnol. 2022 May 30;10:892896. doi: 10.3389/fbioe.2022.892896 (PMC9195575; doi:10.3389/fbioe.2022.892896)
Supplement: Supplementary file 1 [file DataSheet1.PDF]

Supplementary Material

Bioproduction of Linalool from Paper Mill Waste

Mauro A. Rinaldi[1,2], Shirley Tait[2], Helen S. Toogood[1,2], Nigel S. Scrutton[1,2,3*]

[1]Future Biomanufacturing Research Hub and [2]Manchester Institute of Biotechnology, The University of Manchester, 131 Princess Street, Manchester, M1 7DN, United Kingdom

[3]C3 Biotechnologies (Maritime and Aerospace) Ltd, 20 Mannin Way, Caton Road, Lancaster, Lancashire LA1 35W, UK

1. Supplementary Methods

LinS ribosomal binding site modification

The LinS gene is predicted to be one of the bottlenecks in overall linalool production in *E. coli*. The LinS ribosomal binding site in pMVA-GLinS NR2 (RBS; GGATCTTTTAAGAAGGAGATATAC) was analyzed by the RBS Library Calculator (Salis et al., 2009) to predict bases to be randomized to generate alternative sequences that may increase protein expression. A mutagenic library of primers was synthesized (GGA*G*C*B*TTT*T*AGAAGGAG*R*TATAC), where B and R code for C/G/T and A/G, respectively. The pMVA-GLinS NR2 plasmid underwent LinS RBS randomization using the PCR-based Q5 Site-Directed Mutagenesis Kit. Following transformation into *E. coli* DH5α, 30 independent colonies were selected and assayed for linalool production. The top five were sequenced to determine the RBS sequence. Only clone 23 had undergone mutations in the RBS upstream of the LinS gene (aat---GGA*G*CTTTT*T*AGAAGGAG*G*TATA*G*), including the removal of the upstream stop codon generating a GPPS-LinS fusion protein.

Incorporation of neryl pyrophosphate synthase

To increase geranylpyrophosphate biosynthesis, an alternative enzyme neryl pyrophosphate synthase (NPPS) from *Solanum lycopersicum* (GenBank: NM_001247704.1) was synthesized to act as an alternative or additional enzyme to GPPS. The gene was synthesized employing codon optimization for improved expression in *E. coli*. Two PCR amplifications of pMVA-GLinS NR2 were performed where the linearization occurred either upstream of the GPPS gene or leading to the elimination of this gene. The NPPS gene was amplified by PCR and ligated to the linearized plasmids by In-Fusion cloning to generate pMVA-NGLinS NR2 and pMVA-NLinS NR2, respectively (**Table 1**).

Electrocompetent *E. coli* cells

This general protocol of electroporation of *E. coli* is a modification of previously published methods. A starter culture of *E. coli* strain DH5α was cultivated in LB medium containing 150 μg/mL hygromycin overnight at 30 °C. An inoculum (5 mL) was added to 100 mL of the same medium and incubated at 30 °C at 200 rpm for approximately 2 h until the $OD_{600\ nm}$ reached 0.4-0.6. For *E. coli* cells containing the pSIM18 plasmid, there was additional heat treatment

step at this stage to induce the production of the recombination genes. This was performed by incubating the culture at 42 °C for 15 min. All cultures were cooled in ice water for 5-10 min and centrifuged at 3,200 *g* at 4 °C for 10 min. Cells were then washed with ice-cold 10% glycerol, centrifuged as above, and resuspended in ice-cold 10% glycerol. Cells were washed five more times with 1 mL ice-cold 10% glycerol, centrifuging between each wash at 15,000 rpm for 1 min at 4 °C. Cells were resuspended in 100 μL ice-cold 10 % glycerol and stored at -80 °C.

The general protocol for electroporation involved incubating 50 μL of electrocompetent *E. coli* with 300 ng of plasmid DNA or linearized PCR products for 5 min on ice. The slurry was transferred to an electroporation cuvette with a 0.2 mm gap and electroporated at 2.5 kV, 200 Ω, 25 μF. LB recovery medium (500 μL) was added and the culture was incubated at 37 °C for 2 h. Culture aliquots (100 μL) were spread on antibiotic selective LB agar plates and incubated overnight at 37 °C.

Construction of pKIKO vectors with a *gltA E. coli* loci

To have a fourth chromosomal integration site in *E. coli*, we created pKIKO vectors with homology arms for integration into *gltA*, a locus that has high expression in *E. coli* (Yin et al., 2015). pKIKO*gltA*Cm and pKIKO*glta*Km were cloned by amplifying four sections: 1) *gltA* homology arm 1 was amplified from genomic *E. coli* DH5α DNA with primers glta-HA1-F and glta-HA1-R, 2) pKIKO multiple cloning site and selection marker was from amplified from pKIKO*arsB*Cm or pKIKO*arsB*Km with primers pKIKO-MCS-F and pKIKO-MCS-R, 3) *gltA* homology arm 2 was amplified from genomic *E. coli* DH5α DNA with primers glta-HA2-F and glta-HA2-R, 4) pKIKO replication origin was amplified from pKIKO*arsB*Cm with primers pKIKO-ori-F and pKIKO-ori-R. These four sections had 30 bp overlaps and were fused with In-Fusion. The plasmid maps are shown in **Supplementary Figure S1** and primers used for PCR amplification are shown in **Supplementary Table S2**.

Genomic integration using the pKIKO methodology

The general protocol for chromosomal integration is based on the λ-RED recombination system (Thomason et al., 2014) using the pKIKO vector series of plasmids (**Table S1**) (Sabri et al., 2013; Jervis et al., 2021) as described previously. DNA cassettes for integration were inserted within the multiple cloning site of the pKIKO vectors, located upstream of the kanamycin or chloramphenicol resistance gene and between the specific chromosomal loci homology arms (HA1 and HA2). PCR amplification of the pKIKO plasmid generated a linear fragment starting from just outside of each homology arm, which contained the DNA cassette and antibiotic resistance gene. The latter gene was enclosed on each side by the flippase-mediated recombination sequence (FRT) (Sabri et al., 2013). This linear fragment was digested with the restriction enzyme DpnI to eliminate the parental PCR template, followed by electroporation into *E. coli* DH5α containing the pSIM18 plasmid. Cultures were incubated for 2 h at 37 °C, followed by plating (100 μL) onto antibiotic selective LB agar plates. Cultures were incubated overnight at 37 °C, and multiple colonies were analyzed for the presence of the integrated construct by colony PCR. In some cases, individual colonies underwent rounds of replica plating and sub-culturing to eliminate wild-type (non-integrated) contamination. Colonies were checked for the presence of the integrated DNA cassette by PCR, using primers specific to regions outside of the chromosome HA1 and HA2 sites, or with one primer specific for the inserted DNA.

To cure the integrated strains of the pSIM18 plasmid, one colony was inoculated in antibiotic-free LB medium (5 mL) and cultivated at 43 °C for 4-5 h until $OD_{600\ nm}$ = 0.5. Culture aliquots (100 μL) of 10^{-5} and 10^{-6} dilutions were plated on LB agar and incubated overnight at 37 °C. Successful removal of pSIM18 was determined by replica plating 10 colonies on both LB agar and a second plate containing hygromycin. The absence of growth on the hygromycin plate is suggestive of the absence of pSIM18. The elimination of the integrated antibiotic resistance gene flanked by FRT sites was performed using the recombinase flippase system encoded on the heat sensitive plasmid pCP20 (Cherepanov and Wackernagel, 1995; Datsenko and Wanner, 2000). Integrated *E. coli* strains were made electrocompetent and transformed with plasmid pCP20 by electroporation. A single colony was incubated in 5 mL LB medium at 43 °C for 4-5 h until $OD_{600\ nm}$ reached 0.5. This induces both the removal of the pCP20 plasmid and the production of the flippase enzyme, which excises the antibiotic resistance. Successful removal of the antibiotic resistance was determined by replica plating on LB and antibiotic selective LB agar, as above, and colony PCR using primers flanking the FRT sites. Final confirmation of successful gene cassette integration and antibiotic removal was performed by sequencing the genomic DNA in the region of interest.

2. Supplementary Results

Co-expression of a compatible plasmid

We hoped to build on the success of titer improvement and some plasmid stability improvements by applying the environmental stress approach of co-expressing the linalool plasmid (NR1 and NR2) with a second plasmid (pBbB2k-RFP) containing a compatible *ori* and a second antibiotic resistance gene (Gama et al., 2020). As before, high variability in linalool titres was seen (pMVA-GLinS NR1/RFP; 104.5 ± 30.5 mg/L; **Supplementary Figure S3a**), but there was an improvement in full length plasmid maintenance and small colony size was the best indicator for high linalool titres (**Supplementary Figure S3b**).

Plasmid engineering of LinS and GPPS

Another approach to increasing linalool titers is to target the two latter enzymes in the pathway (GPPS and LinS) for modification. Prior studies showed that higher linalool titers can be attained with the double variant $LinS_{L72M/V214I}$ (Ferraz et al., 2021). These residues were originally chosen for mutagenesis in an effort to minimize the production of the contaminating monoterpenoid nerolidol, generated by the action of LinS on farnesyl pyrophosphate (FPP) (Karuppiah et al., 2017). This double variant previously showed monoterpenoid titers up to 1 g/L organic overlay, with a linalool content of more than 90% (Ferraz et al., 2021). A second variant was generated where the first 29 amino acids of the chloramphenicol acetyltransferase enzyme was added to the N-terminus of LinS (pMVA-G-CmR29*LinS NR2) to enhance its soluble expression in *E. coli* (Wang et al., 2019). We tested the plasmid stability of the $LinS_{L72M/V214I}$ and CmR29*LinS variants in the NR2 backbone by performing linalool assays and diagnostic restriction digests (**Supplementary Figure S5a-b**). Unfortunately, in both cases the linalool titers were decreased compared to the pMVA-GLinS NR2 construct, with very little evidence of intact plasmid remaining after growth for both variants.

An attempt to improve the expression of LinS by modifying its RBS sequence generated a variant (RBS 23) that was missing the stop codon at the end of the upstream GPPS gene. This generated a fusion GPPS-LinS gene with a small linker region. This is similar to prior studies that generated a fusion between GPPS and pinene synthase (Sarria et al., 2014), leading to higher titers of pinene. Linalool production of the fusion GPPS-LinS construct showed linalool

titers of around 210 mg/L (**Supplementary Figure S5c**). In addition, linalool production of a pMVA-GLinS NR2 construct with a mutated RBS (RBS 11) showed similar linalool titers to the GPPS-LinS construct. In another report, fusions of LinS-GPPS did not lead to elevation in the titers of linalool (Wang et al., 2019).

A recent study showed the production of the related monoterpenoid limonene was enhanced by a substitution of GPPS for the N-terminally truncated neryl pyrophosphate synthase (NPPS) from tomato (*Solanum lycopersicum*) (Wu et al., 2019). This was tested for linalool production by either substituting GPPS for NPPS (pMVA-NLinS NR2) or co-expressing it with GPPS (pMVA-NGLinS NR2). Unfortunately, in both cases there was a significant decrease in linalool titers compared to the comparable GPPS-containing construct (**Supplementary Figure S5d**). Overall, studies with the plasmid-born constructs have shown that further optimizations are required to increase both the linalool titers and intact plasmid retention before stable and reproducible titers can be obtained.

Growth on cellulose-based carbon sources

We investigated the growth of *E. coli* BL21(DE3) on a variety of cellulose-based carbon sources and compared them to growth on glucose (**Supplementary Figure S8)**. To reduce the chance of microbial contamination introduced via the addition of the cellulase blend, *E. coli* strain BL21(DE3) was transformed with pBbS5a:RFP plasmid and cultivated in the presence of carbenicillin. In a secondary approach, we tried a consolidated bioprocessing approach (Banner et al., 2021) where we expressed the pCellulose plasmid containing two *E. coli* expression optimized cellulases capable of releasing glucose from cellulose (Bokinsky et al., 2011). Due to the opaqueness of some of the cellulose carbon sources (e.g. paper), growth was monitored by determining the colony forming units (CFU) after 24 h cultivation. Interestingly, significant growth was detected in the cultures containing the added cellulase blend in the absence of additional carbon source. This is likely because some commercial cellulase blends contain added sugar. Growth on carboxymethyl cellulose (CMC), Sigmacell cellulose and paper was highest in the presence of the added cellulase blend, even after subtracting growth due to simple carbon source content (**Supplementary Figure S8**). These levels were significantly higher than cultures expressing pCellulose enzymes or even growth on glucose. The small amount of growth on paper in the absence of any cellulase enzymes is a reflection on the complex nature of paper and the presence of *E. coli* BL21(DE3) on multiple potential carbon sources in situ. Given the high growth of *E. coli* on paper secondary fiber supplemented with the cTec2 cellulase blend, we decided to take this forward to demonstrate linalool production from cellulose-based carbon sources.

3. Supplementary Figures

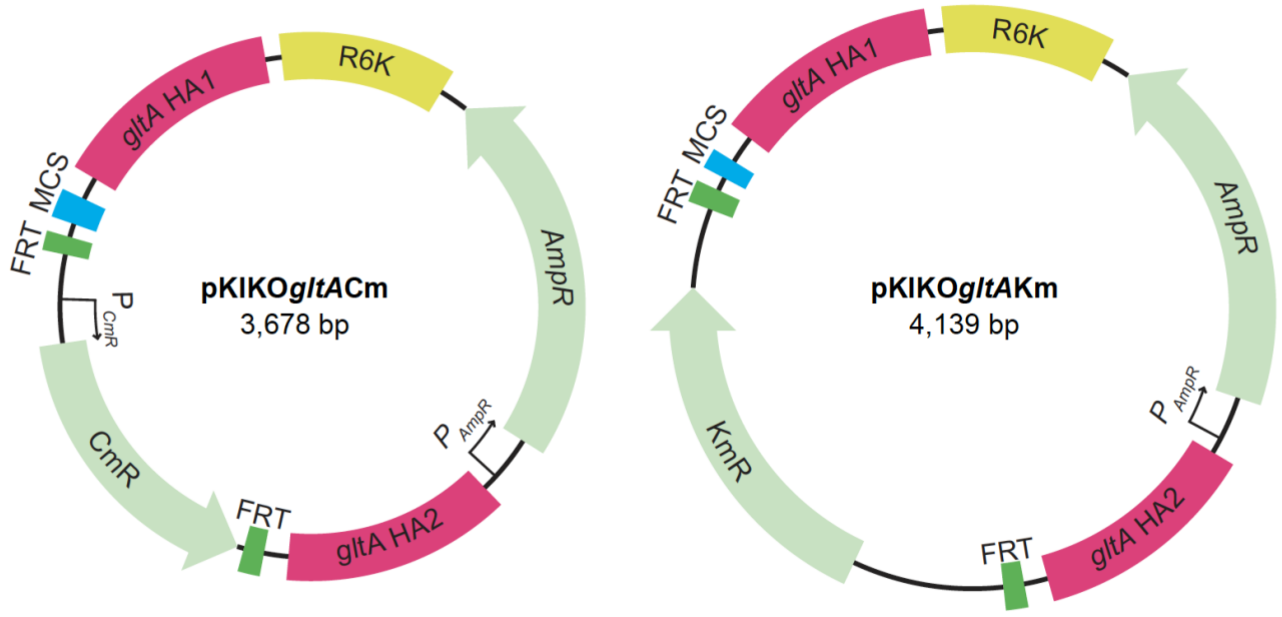

Figure S1. Plasmids maps of A) pKIKO*gltA*Cm and B) pKIKO*glta*Km for the integration of recombinant DNA into the *gltA* loci of *E. coli* (Yin et al., 2015).

A)

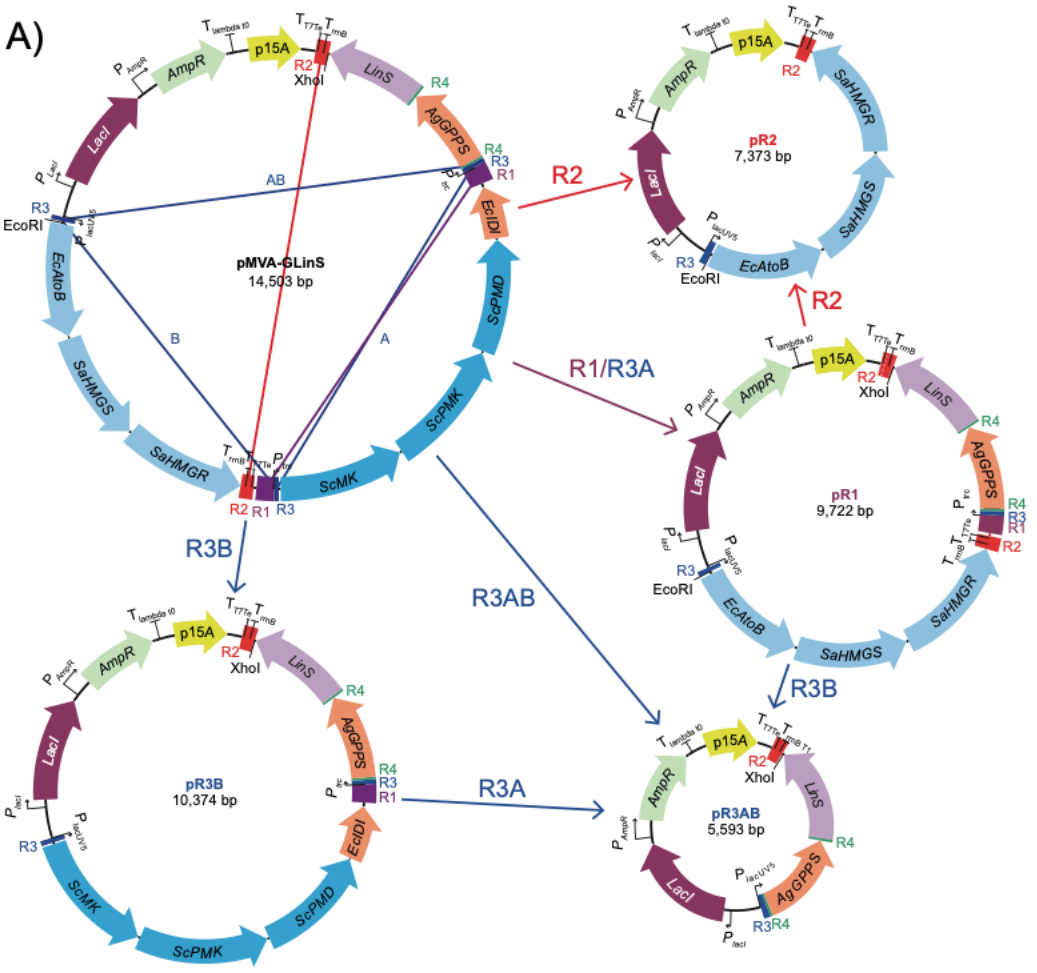

B)

Plasmid sizes after digestion with EcoRI and XhoI.

pMVA-GLinS	Original	pR1/R3A	pR2	pR3B	pR3AB	pR4
Original	10851 + 3652	6070 + 3652	7373	10374	5593	(-918)
NR1	10504 + 3652	5723 + 3652	-	10374	5593	(-918)
NR2	10480 + 3652	-	-		5593	(-918)

Figure S2. Diagnostic restriction enzyme test for pMVA-GLinS plasmid recombination. A) Predicted recombination events and resultant plasmids. B) Predicted molecular mass of digested DNA fragments of pMVA-GLinS and recombination products. Plasmid DNA was purified after each 68-72 h linalool assay, and digested with EcoRI and XhoI, and the fragments were analyzed by electrophoresis using a 0.5% agarose gel. (-918) = recombination of any plasmid, except pR2, can result in losing an additional 918 bp.

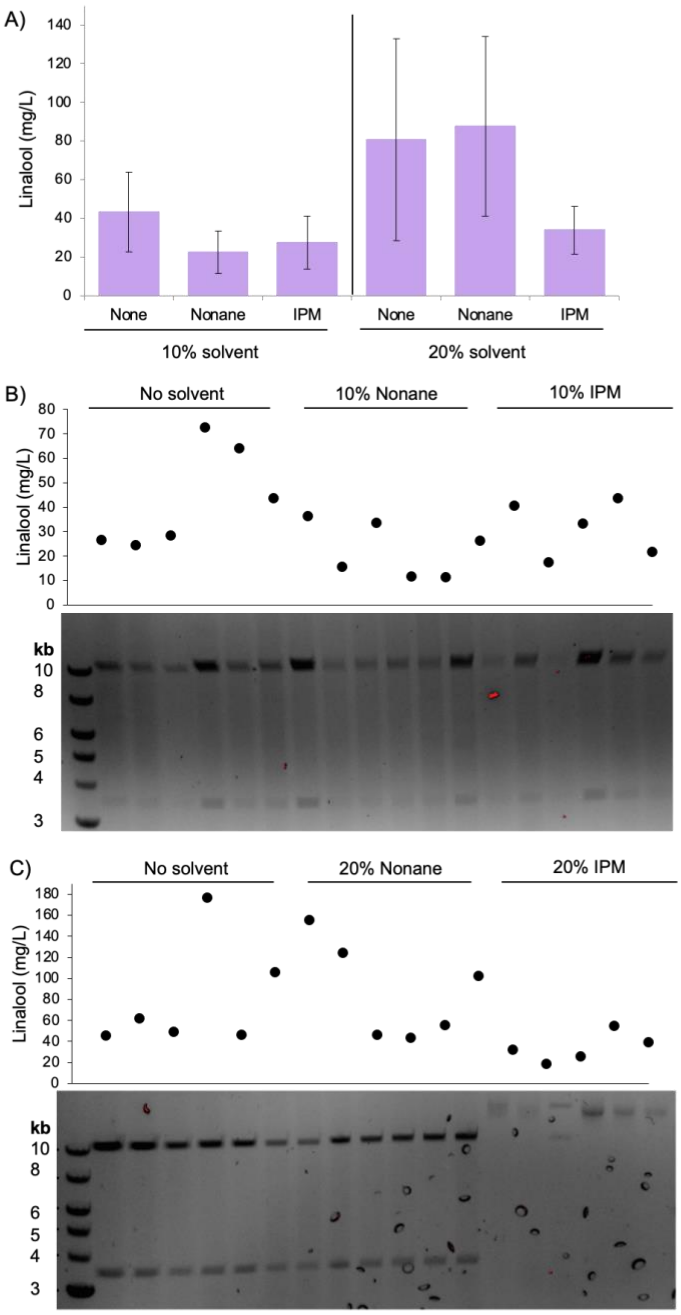

Figure S3. Linalool production by *E. coli* DH5α containing pMVA-GLinS NR1/NR2 with a second compatible plasmid. A) Spread of linalool titers and B) plasmid size comparison among biological replicates. Cultures (3 mL) were incubated in TB medium containing 0.4% glucose and antibiotic selection at 37 °C until growth was visible, followed by induction with 50 µM IPTG. A further incubation at 30 °C was performed for 68-72 h and linalool production was determined by GCMS analysis. The plasmid diagnostic gel lanes are aligned horizontally with the linalool data points (black circles), which are individual linalool assays of single colonies. Error bars represent one standard deviation of the data. Individual biological replicates are shown as black spheres.

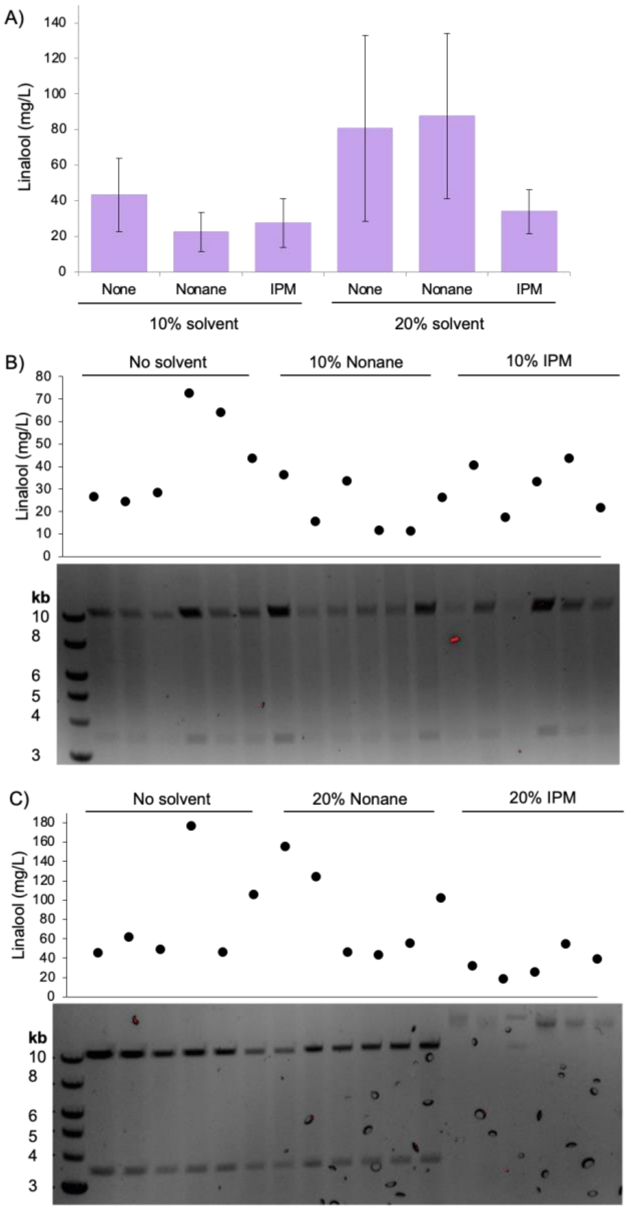

Figure S4. Linalool production by *E. coli* DH5α containing pMVA-GLinS NR2 in the presence of an organic solvent overlay. A) Summary of linalool titers in the presence of B) 10% and C) 20% nonane or isopropyl myristate (IPM). Cultures (3 mL) were incubated in TB medium containing 0.4% glucose and antibiotic selection at 37 °C until growth was visible, followed by induction with 50 µM IPTG and the addition of the organic overlay (10-20%). A further incubation at 30 °C was performed for 68-72 h and linalool production was determined by GCMS analysis. Individual biological replicates are shown as black spheres and the plasmid recombination diagnostic agarose gel electrophoresis of each assay culture is shown below each sample. The plasmid diagnostic gel lanes are aligned horizontally with the linalool data points (black circles), which are individual linalool assays of single colonies.

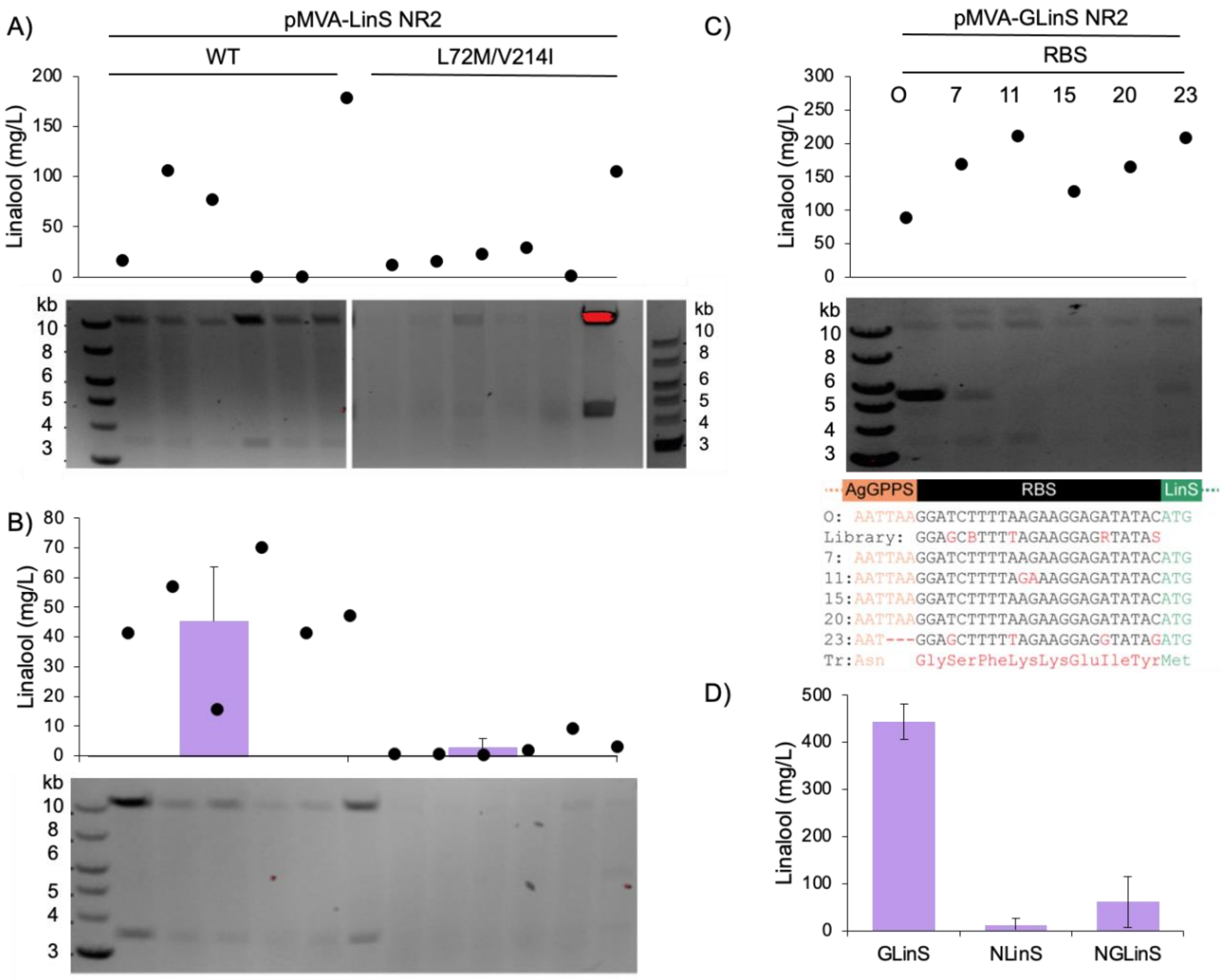

Figure S5. Linalool production by *E. coli* DH5α containing variants of the one-plasmid system. A) Effect of LinS variant L72M/V214I double variant on linalool production. B) Effect of the addition of an N-terminal chloramphenicol-resistance solubility tag (Wang et al., 2019) on LinS on linalool production. C) Effect of alterations in the RBS between GPPS and LinS on linalool titers. RBS 23 is missing the stop codon, so generates a GPPS-LinS fusion protein. The lower panel shows the sequence changes in the RBS screen. D) Effect of the substitution of GPPS for neryl pyrophosphate synthase (NPPS) (Wu et al., 2019) or its inclusion with GPPS on linalool production. Cultures (3 mL) were incubated in TB medium containing 0.4% glucose and antibiotic selection at 37 °C until growth was visible, followed by induction with 50 µM IPTG. A further incubation at 30 °C was performed for 68-72 h and linalool production was determined by GCMS analysis. Individual biological replicates are shown as black spheres and the plasmid recombination diagnostic agarose gel electrophoresis of each assay culture is shown below each sample. The plasmid diagnostic gel lanes are aligned horizontally with the linalool data points (black circles), which are individual linalool assays of single colonies.

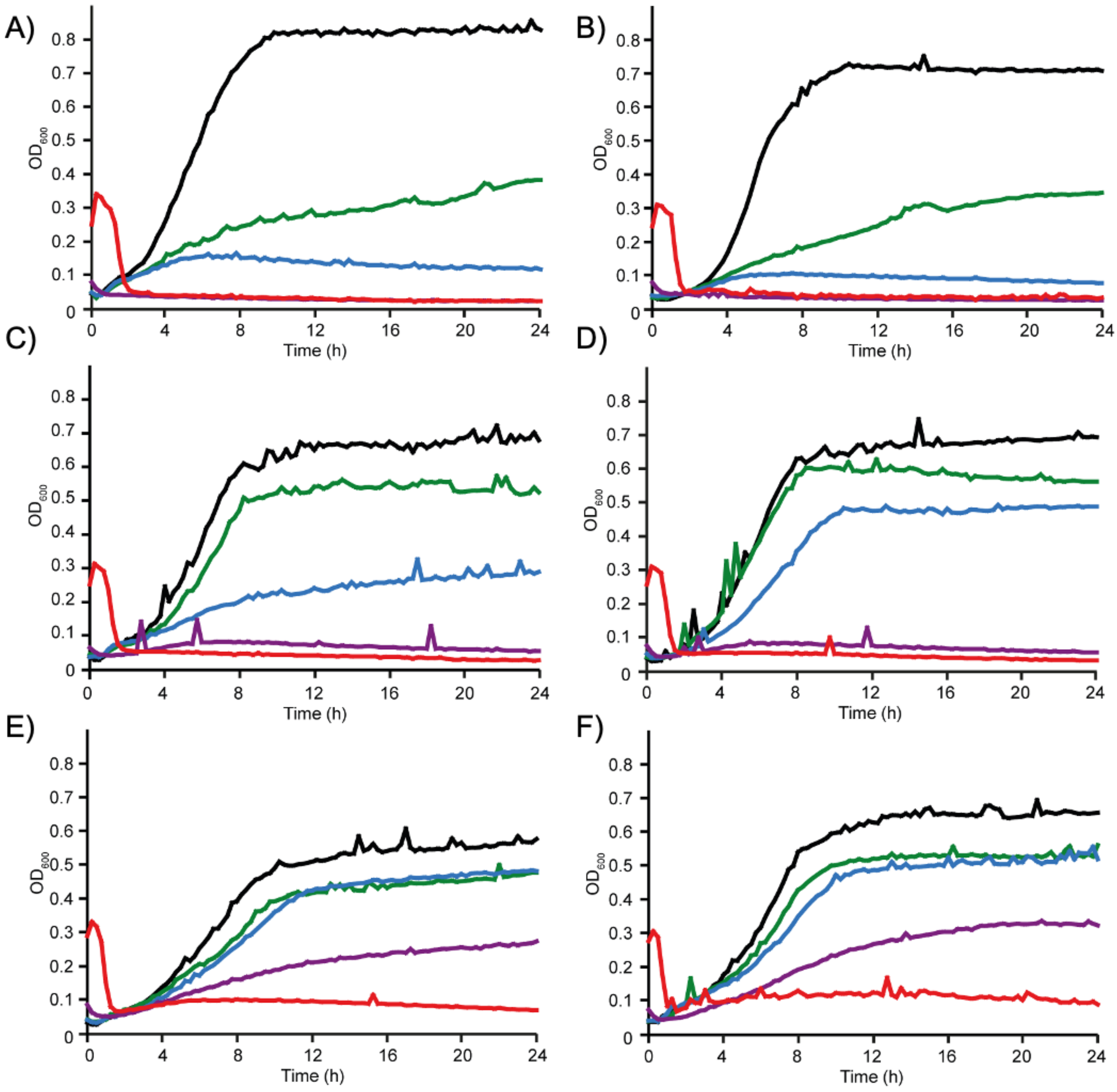

Figure S6. Growth of A) wild-type *E. coli* DH5a and adapted laboratory evolution (ALE) strains ALE-1 B), ALE-2.5 C), ALE4 D), ALE-10 E) and ALE-20 F) in the presence of linalool. Replicate aliquots of an overnight *E. coli* DH5α culture (200 μL) were set up in LB medium containing 0-20 g/L linalool in a 96-well microtiter plate covered with a Moisture Barrier Seal and incubated at 30 °C with 500 rpm agitation within a FLUOstar Omega Microplate reader. Optical density readings ($OD_{600\ nm}$) were performed every 15 minutes for 24 h. Growth curves for linalool concentrations of 0, 2, 5, 10 and 20 g/L are shown as black, green, blue, magenta, and red lines, respectively.

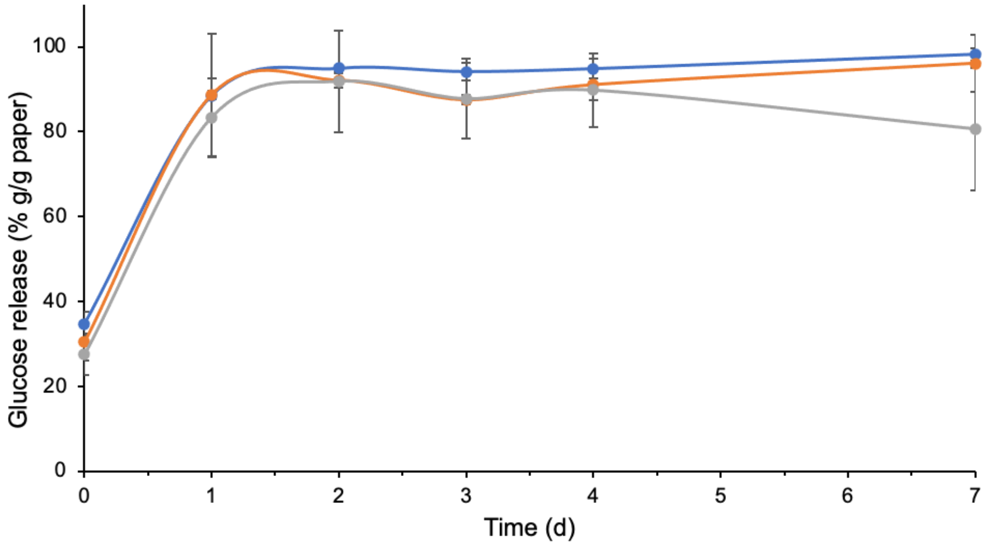

Figure S7. Glucose release from enzymatic hydrolysis of paper samples. Glucose release was performed by an adaptation of the NREL procedure (Resch et al., 2015) using Novozymes Cellic cTec2 cellulase enzyme blend. Error bars represent standard deviations from triplicates. Paper samples: Blue = commercial paper sample; orange = alkali pretreated JK Paper Ltd wastewater paper fines and grey = acid pretreated JK Paper Ltd wastewater paper fines.

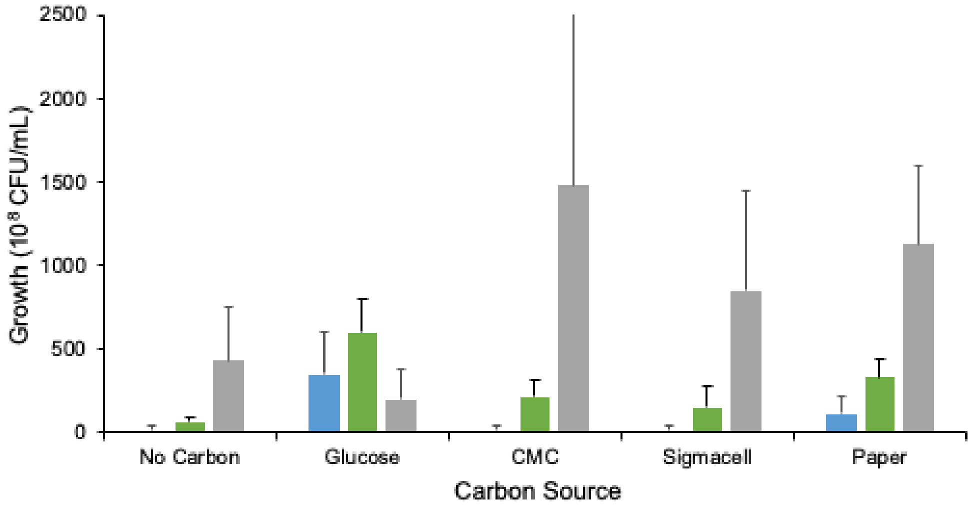

Figure S8. Cultivation of *E. coli* BL21(DE3) using cellulose-based carbon sources. Colony forming units of *E. coli* cultivated in minimal medium containing pre-treated paper samples (saccharified for 48 h) as the carbon source. Simultaneous saccharification and fermentation was performed in LB medium by the addition of the Cellic cTec2 cellulase blend (1:100) or expression of the ampicillin-resistant pCellulose multi-cellulase plasmid (Bokinsky et al., 2011). Cellulase enzyme source: blue = empty vector (no saccharification); green = pCellulose plasmid and grey = cTec2 enzyme blend. Error bars represent the standard deviations of triplicate cultivations.

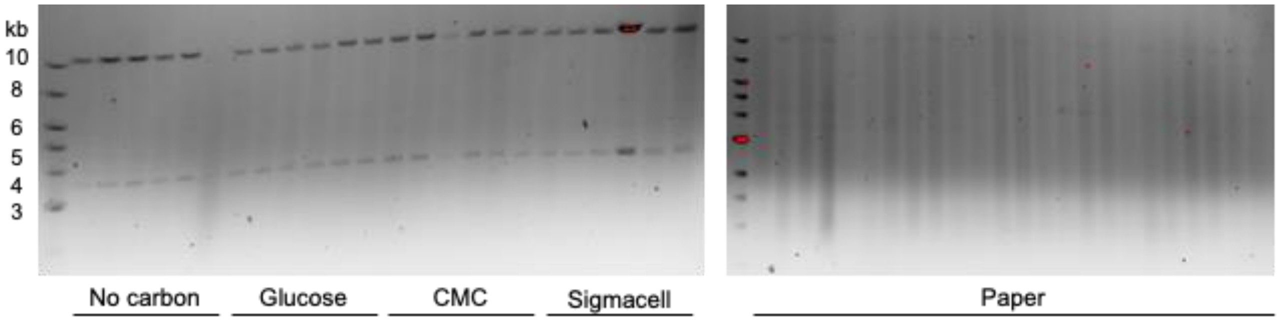

Figure S9. Diagnostic restriction digest and agarose gel electrophoresis of the pMVA-GLinS NR2 plasmid isolated from *E. coli* DH5α cultures cultivated on cellulose-based carbon sources. Cultures were grown on minimal medium containing a sole carbon source. Glucose release from cellulose-based substrates was facilitated by performing simultaneous saccharification and fermentation via the addition of the cTec2 enzyme blend. Following cultivation, plasmids were extracted, purified and digested with EcoRI and XhoI. The presence of a full-length (non-recombined) plasmid is inferred by the presence of a 10 kb band.

4. Supplementary Tables

Table S1. Plasmids used in this study for the construction of linalool production pathway plasmids and to facilitate genomic integration of DNA.

Plasmid	Description[1]	Source/Reference
Plasmid backbones and intermediate constructs to generate genome integration cassettes		
pKIKO*arsB*Cm:GLinS	R6K, Cm^R, P_{Tet}-GLinS-T_{rrnBT1}-T_{T7TE}	This work
pKIKO*lacZ*Cm:MevB	R6K, Cm^R, P_{trc}-MevB-T_{rrnBT1}-T_{T7TE}	This work
pKIKO*rbsAR*Cm:MevT	R6K, Cm^R, P_{lacUV5}-MevT-T_{rrnBT1}-T_{T7TE}	This work
pKIKO*gltA*Cm	R6K, Cm^R	This work
pKIKO*gltA*Km	R6K, Kan^R	This work
Plasmids used to facilitate DNA cassette integration into the E. coli genome		
pSIM18	SC101, Hyg^R, P_{RM}-cI857, P_L-*exo*,*bet*,*gam* (λ-RED)	Chan *et al*., 2007
pCP20	OriR101/*repA101ts*, Amp^R, FLP recombinase	Cherepanov and Wackernagel, 1995

[1]Replication origin, antibiotic marker and promoter-operon-terminator. [2]Flanked by homology sequences to replace the P_{trc} promoter in the chromosome. λ-RED = λ-RED recombination genes.

Table S2. Primers used in this study

Name	Sequence	Purpose
pKIKO-linear-F<br>pKIKO-linear-R	GCATTTATCAGGGTTATTGTCTCATGAGCG<br>GAATTAGCCATGGTCCATATGAATATCCTCCC	Linearize pKIKO plasmids for chromosomal integration.
pML-NR1-F<br>pML-NR1-R	ATCTTGACAATTAATCATCCGGCTCGTATAATG<br>TGTGGAATTGTGAG<br>CGGATAACAATTTCAGGATCTAGG<br>TATACGAGCCGGATGATTAATTGTCAAGATCCTT<br>ATTGTTGTCTAATTT CTTGTAAAATATGTTCCGC	Amplify pMVA-GLinS NR1 without repeated region R1, and smaller R2.
pML-NR2-F<br>pML-NR2-R	CTAGCTCAGTCCTAGGGACTATGCTAGCGGATCT<br>AGGAGGAAAT AACCATGTCTCTGCCATTCC<br>TAGTCCCTAGGACTGAGCTAGCTGTCAAGATCCT<br>TATTGTTGTCTAAT<br>TTCTTGTAAAATATGTTCCGC	Amplify pMVA-GLinS NR2 without repeated regions R1, R2 and R3, and replacing the *trc* promoter with the *J23116* promoter.
GLinS-SalI-F<br>GLinS-SacI-R	CTGACGTCGACCATTTCCCCGAAAAGTGCCACC<br>GAATACCGAGCTCAGCAACCCGTAAGTGCGC	Amplify GLinS from pGLinS for cloning into pKIKO to create pKIKO*arsB*Cm:GLinS.
MevB-F<br>MevB-R	TCATATAGACCATGACTGCATAATTCGTGTCGCT<br>CAAGG<br>CAGGAATTCCATATGGCGGAATATATCCCTAGGT<br>ATAAACGCAG	Amplify MevB from pMVA into pKIKO to create pKIKO*lacZ*Cm:MevB.
pKIKO-F<br>pKIKO-R	CATATGGAATTCCTGCAGTGCGC<br>TCATGGTCTATATGAATATCCTCCTTAGTTCCTAT<br>TCC	Amplify pKIKO vectors to insert constructs of interest.
MevT-SalI-F<br>MevT-SacI-R	GTGACGTCGACCTGCATAATTCGTGTCGCTCAAG<br>G<br>GAATTGCGAGCTCGCGGAATATATCCCTAGGTAT<br>AAACGCAG	Amplify MevT from pMVA for cloning into pKIKO to create pKIKO*rbsAR*Cm:MevT.
gltA-HA1-F<br>gltA-HA1-R	TATCCTCCCAGAAACTCGAAGGTTACGCCAACTT<br>TGGATACC<br>GTTTTTTTCGCTGTTTAACCCTATGAAAATGACGT<br>CTATCTATACCCCC	Amplify homology arm 1 downstream of gltA to create pKIKO*gltA*Cm and pKIKO*gltA*Km.
pKIKO-MCS-F<br>pKIKO-MCS-R	CATTTTCATAGGGTTAAACAGCGAAAAAACCCC<br>GCCC<br>GGTAGTCGCATTTATAAACAAAAAAAACCCCGC<br>TTCGGCG	Amplify pKIKO resistance gene and multiple cloning site to create pKIKO*gltA*Cm and pKIKO*gltA*Km.
gltA-HA2-F<br>gltA-HA2-R	GGGTTTTTTTTGTTTATAAATGCGACTACCATGA<br>AGTTTTTAATTGAAAGT ATTGGG<br>CCTGCATCCAGAAACCTGAAAGAGCTGGGCACG<br>AAGG	Amplify homology arm 2 downstream of gltA to create pKIKO*gltA*Cm and pKIKO*gltA*Km.
pKIKO-ori-F<br>pKIKO-ori-R	GCCCAGCTCTTTCAGGTTTCTGGATGCAGGTGGC<br>ACTTTTCG<br>TGGCGTAACCTTCGAGTTTCTGGGAGGATATTCA<br>TATGGACCATGG	Amplify pKIKO origin to create pKIKO*gltA*Cm and pKIKO*gltA*Km.
pKIKO-linear-F<br>pKIKO-J-linear-R	GCATTTATCAGGGTTATTGTCTCATGAGCG<br>GGGGCGGGGTTTTTTGCG	Linearize pKIKO*arsB*Cm:J23100.
pMG-F<br>pMG-R	GTATATCTCCTTCTTAAAAGATCCTTAATTCTGA<br>CGAAATGCC<br>GGATCCAAACTCGAGTAAGGATCTCCAGG	Clone synthases into pMVA-GLinS to make pMVA-GLinS$_{L72M}$ NR2 and pMVA-GLinS$_{L72M/V214I}$ NR2.
LinSv-F	CTTTTAAGAAGGAGATATACATGCAAGAATTTG<br>AATTTGCGGTTCCG	Amplify LinS variants to clone into pMVA-GLinS to make pMVA-GLinS$_{L72M}$ NR2 and pMVA-GLinS$_{L72M/V214I}$ NR2.

LinSv-R	CCTTACTCGAGTTTGGATCCTTAACCGCTGCTAC GTGCCAC	
330-NPPS-F<br>331-NPPS-R	cttttaagaaggagatatacATGAGCAGCCTGGTTCTGCAG<br>GCAAATTCAAATTCTTGCATgtatatctccttcttaaaagatcc TTAATAGGTATGACCACC	Amplify pMVA-GLinS NR2 for cloning to make pMVA-NLinS NR2 and pMVA-NGLinS NR2.
optRBSLinS-F<br>optRBSLinS-R	GGAGCBTTTTAGAAGGAGRTATASATGCAAGAA TTTGAATTTGCGGT TCCG<br>TTAATTCTGACGAAATGCCACGTAGTCTGC	Make RBS library for LinS RBS optimisation.
rGPPS-F	gtatatctccttcttaaaagatcctg	Amplify pMVA-LinS NR2 to replace/add NPPS.
rGPPS-R	ATGCAAGAATTTGAATTTGCgGTTCCG	Amplify pMVA-LinS NR2 without GPPS to replace with NPPS.
NPPS-F	cttttaagaaggagatatacATGAGCAGCCTGGTTCTGCAG	Amplify NPPS to replace/add pMVA-LinS NR2
NPPS-R	GCAAATTCAAATTCTTGCATgtatatctccttcttaaaagatcc TTAATAGGTATGACCACC	Amplify NPPS to replace GPPS in pMVA-LinS NR2.
NPPS-GPPS-R	tggaatttgacttcaacaaatacatggactcc	Amplify pMVA-LinS NR2 to add NPPS.
NPPS-GPPS-R2	tttgttgaagtcaaattccatgtatatctccttcttaaaagatccTTAATAGG TATGACCACC	Amplify NPPS to add to pMVA-LinS NR2

5. References

Banner, A., Toogood, H.S., and Scrutton, N.S. (2021). Consolidated bioprocessing: Synthetic biology routes to fuels and fine chemicals. *Microorganisms* 9(5)**,** 1079. doi:10.3390/microorganisms9051079

Bokinsky, G., Peralta-Yahya, P.P., George, A., Holmes, B.M., Steen, E.J., Dietrich, J., et al. (2011). Synthesis of three advanced biofuels from ionic liquid-pretreated switchgrass using engineered *Escherichia coli*. *Proc. Natl. Acad. Sci. U. S. A.* 108(50)**,** 19949-19954. doi:10.1073/pnas.1106958108.

Cherepanov, P.P., and Wackernagel, W. (1995). Gene disruption in *Escherichia coli*: TcR and KmR cassettes with the option of Flp-catalyzed excision of the antibiotic-resistance determinant. *Gene* 158(1)**,** 9-14. doi:10.1016/0378-1119(95)00193-A.

Datsenko, K.A., and Wanner, B.L. (2000). One-step inactivation of chromosomal genes in *Escherichia coli* K-12 using PCR products. *Proc. Natl. Acad. Sci. U. S. A.* 97(12)**,** 6640. doi:10.1073/pnas.120163297.

Ferraz, C.A., Leferink, N.G.H., Kosov, I., and Scrutton, N.S. (2021). Isopentenol utilization pathway for the production of linalool in *Escherichia coli* using an improved bacterial linalool/nerolidol synthase. *ChemBioChem* 22(13)**,** 2325-2334. doi:10.1002/cbic.202100110.

Gama, J.A., Zilhão, R., and Dionisio, F. (2020). Plasmid interactions can improve plasmid persistence in bacterial populations. *Front. Microbiol.* 11**,** 2033. doi:10.3389/fmicb.2020.02033.

Jervis, A.J., Hanko, E.K.R., Dunstan, M.S., Robinson, C.J., Takano, E., and Scrutton, N.S. (2021). A plasmid toolset for CRISPR-mediated genome editing and CRISPRi gene regulation in *Escherichia coli*. *Microb. Biotechnol.* 14(3)**,** 1120-1129. doi:10.1111/1751-7915.13780.

Karuppiah, V., Ranaghan, K.E., Leferink, N.G., Johannissen, L.O., Shanmugam, M., Ní Cheallaigh, A., et al. (2017). Structural basis of catalysis in the bacterial monoterpene synthases linalool synthase and 1, 8-cineole synthase. *ACS Catal.* 7(9)**,** 6268-6282. doi:10.1021/acscatal.7b01924.

Resch, M.G., Baker, J.O., and Decker, S.R. (2015). *Low solids enzymatic saccharification of lignocellulosic biomass: Laboratory Analytical Procedure (LAP).* Golden, CO, USA: National Renewable Energy Laboratory.

Sabri, S., Steen, J.A., Bongers, M., Nielsen, L.K., and Vickers, C.E. (2013). Knock-in/Knock-out (KIKO) vectors for rapid integration of large DNA sequences, including whole metabolic pathways, onto the *Escherichia coli* chromosome at well-characterised loci. *Microb. Cell Fact.* 12(1)**,** 60. doi:10.1186/1475-2859-12-60.

Salis, H.M., Mirsky, E.A., and Voigt, C.A. (2009). Automated design of synthetic ribosome binding sites to control protein expression. *Nat. Biotechnol.* 27(10)**,** 946-950. doi:10.1038/nbt.1568.

Sarria, S., Wong, B., Martín, H.G., Keasling, J.D., and Peralta-Yahya, P. (2014). Microbial synthesis of pinene. *ACS Synth. Biol.* 3(7)**,** 466-475. doi:10.1021/sb4001382.

Thomason, L.C., Sawitzke, J.A., Li, X., Costantino, N., and Court, D.L. (2014). Recombineering: Genetic engineering in bacteria using homologous recombination. *Curr. Protoc. Mol. Biol.* 106(1)**,** 1.16.11-11.16.39. doi:10.1002/0471142727.mb0116s106.

Wang, X., Wu, J., Chen, J., Xiao, L., and Li, X. (2019). Efficient biosynthesis of *R*-(-)-linalool through adjusting expression strategy and increasing GPP supply in *Escherichia coli*. *J. Agric. Food Chem.* 68(31)**,** 8381-8390. doi:10.1021/acs.jafc.0c03664.

Wu, J., Cheng, S., Cao, J., Qiao, J., and Zhao, G.-R. (2019). Systematic optimization of limonene production in engineered *Escherichia coli*. *J. Agric. Food Chem.* 67(25)**,** 7087-7097. doi:10.1021/acs.jafc.9b01427.

Yin, J., Wang, H., Fu, X.-Z., Gao, X., Wu, Q., and Chen, G.-Q. (2015). Effects of chromosomal gene copy number and locations on polyhydroxyalkanoate synthesis by *Escherichia coli* and *Halomonas* sp. *Appl. Microbiol. Biotechnol.* 99(13)**,** 5523-5534. doi:10.1007/s00253-015-6510-8.
